# NopD of *Bradyrhizobium* sp. XS1150 Possesses SUMO Protease Activity

**DOI:** 10.3389/fmicb.2020.00386

**Published:** 2020-03-20

**Authors:** Qi-Wang Xiang, Juan Bai, Jie Cai, Qin-Ying Huang, Yan Wang, Ying Liang, Zhi Zhong, Christian Wagner, Zhi-Ping Xie, Christian Staehelin

**Affiliations:** State Key Laboratory of Biocontrol and Guangdong Key Laboratory of Plant Resources, School of Life Sciences, Sun Yat-sen University, Guangzhou, China

**Keywords:** effector, legume, nitrogen fixation, nodulation, protease, small ubiquitin-related modifier, symbiosis, type III protein secretion system

## Abstract

Effectors secreted by the type III protein secretion system (T3SS) of rhizobia are host-specific determinants of the nodule symbiosis. Here, we have characterized NopD, a putative type III effector of *Bradyrhizobium* sp. XS1150. NopD was found to possess a functional N-terminal secretion signal sequence that could replace that of the NopL effector secreted by *Sinorhizobium* sp. NGR234. Recombinant NopD and the C-terminal domain of NopD alone can process small ubiquitin-related modifier (SUMO) proteins and cleave SUMO-conjugated proteins. Activity was abolished in a NopD variant with a cysteine-to-alanine substitution in the catalytic core (NopD-C_972_A). NopD recognizes specific plant SUMO proteins (AtSUMO1 and AtSUMO2 of *Arabidopsis thaliana*; GmSUMO of *Glycine max*; PvSUMO of *Phaseolus vulgaris*). Subcellular localization analysis with *A. thaliana* protoplasts showed that NopD accumulates in nuclear bodies. NopD, but not NopD-C_972_A, induces cell death when expressed in *Nicotiana tabacum*. Likewise, inoculation tests with constructed mutant strains of XS1150 indicated that nodulation of *Tephrosia vogelii* is negatively affected by the protease activity of NopD. In conclusion, our findings show that NopD is a symbiosis-related protein that can process specific SUMO proteins and desumoylate SUMO-conjugated proteins.

## Introduction

Various bacteria possess protein secretion systems, through which effectors are translocated into host cells. Type III (T3) effector proteins secreted via a needle-like type III secretion system (T3SS) are important virulence factors of pathogenic bacteria such as the plant pathogens *Pseudomonas syringae* and *Xanthomonas campestris* (Büttner, 2016; Deng et al., 2017). Functional T3SS have also been identified in various rhizobia, bacteria that establish a symbiotic relationship with legumes (Staehelin and Krishnan, 2015; Nelson and Sadowsky, 2015; López-Baena et al., 2016). Rhizobia, differentiated into bacteroids, reduce atmospheric nitrogen to ammonia in root nodules of host plants. Fixed nitrogen is delivered to the host plant in exchange of carbon assimilates and nutrients. Consequently, growth of legume crops does not depend on application of nitrogen fertilizer. Rhizobial infection and nodule initiation are controlled by various signals, including host flavonoids and rhizobial lipo-chitooligosaccharides, the so-called Nod factors (Perret et al., 2000; Oldroyd, 2013; Ferguson et al., 2019). Mutant analysis showed that several rhizobial T3 effectors of various strains also play a crucial role in establishment and maintenance of the symbiosis (Staehelin and Krishnan, 2015; Nelson and Sadowsky, 2015; López-Baena et al., 2016). However, besides secretion and translocation into host cells, only a few rhizobial T3 effectors have been biochemically characterized in detail. Examples of well-studied rhizobial effectors are the nodulation outer proteins NopE1/NopE2 (Wenzel et al., 2010; Schirrmeister et al., 2011), NopL (Bartsev et al., 2003; Bartsev et al., 2004; Zhang et al., 2011; Ge et al., 2016), NopM (Rodrigues et al., 2007; Kambara et al., 2009; Xin et al., 2012; Xu et al., 2018), NopP (Ausmees et al., 2004; Skorpil et al., 2005; Zhao et al., 2018; Sugawara et al., 2018), NopT (Dai et al., 2008; Dowen et al., 2009; Kambara et al., 2009; Fotiadis et al., 2012), and ErnA (Teulet et al., 2019).

Pattern recognition receptors of plants recognize structurally conserved microbial elicitors (PAMPs) to activate defense gene expression. In most cases, PAMP recognition results in PTI (Boller and Felix, 2009; Macho and Zipfel, 2014; Cao et al., 2017). T3 effectors translocated into plant cells often suppress PTI and some of them target pattern recognition receptors and downstream signaling components such as mitogen activated protein (MAP) kinases (Feng and Zhou, 2012). The T3 effector NopL of *Sinorhizobium* sp. (=*Ensifer fredii*) NGR234, for example, becomes multiply phosphorylated by MAP kinases and thereby inhibits MAP kinase signaling (Zhang et al., 2011; Ge et al., 2016). On the other hand, plants can recognize the presence or action of a specific T3 effector (avirulence protein) by a given intracellular disease resistance protein (nucleotide-binding/leucine-rich repeat receptor). This triggers a rapid and strong defense reaction that often culminates in programmed cell death, the so-called hypersensitive response. In this way, growth of invading pathogens is rapidly arrested and the T3 effector functions as an avirulence protein (ETI) (Cui et al., 2015). A strong hypersensitive response was also observed when the rhizobial effector protease NopT was expressed in the non-host plant tobacco (*Nicotiana tabacum*) (Dai et al., 2008; Fotiadis et al., 2012). Likewise, NopT and other rhizobial effectors (Staehelin and Krishnan, 2015) have a negative impact on nodule formation in certain host plants. In soybean (*Glycine max*), special forms of the disease resistance protein Rj2 are involved in blockage of nodule formation by specific *Bradyrhizobium* and *Sinorhizobium* strains in a T3SS-dependent manner (Yang et al., 2010; Sugawara et al., 2018). ETI-like defense responses were observed in a specific soybean cultivar (*Rj4/Rj4* genotype) inoculated with *B. elkanii* USDA61 (Yasuda et al., 2016). Positional cloning revealed that the *Rj4* gene encodes a thaumatin-like protein (Tang et al., 2016). Nodulation tests with rhizobia mutagenized with the Tn5 transposon indicated that *Rj4*-mediated nodulation blockage can be overcome by deletion of a putative T3 effector gene (*BEL2_5* in USDA61, Faruque et al., 2015; *MA20_12780* in *B. japonicum* Is-34, Tsurumaru et al., 2015).

Post-translational ubiquitination of proteins followed by degradation via the ubiquitin proteasome system regulates protein levels in eukaryotic cells. To suppress PTI, T3 effectors can interfere with the ubiquitin proteasome system. For example, T3 effectors of pathogenic bacteria can mimic the activity of ubiquitin ligases and therefore label PTI-related host proteins for proteasome-dependent degradation (Dudler, 2013; Banfield, 2015). Likewise, the E3 ubiquitin ligase NopM, a T3 effector of *Sinorhizobium* sp. NGR234, can dampen PAMP-induced generation of reactive oxygen species in *Nicotiana benthamiana* cells (Xin et al., 2012). Besides the ubiquitin system, effectors delivered to host cells may interfere with sumoylation, i.e. conjugation of a protein to a small ubiquitin-like modifier (SUMO) protein. Sumoylation in eukaryotic cells regulates various processes such as transcriptional regulation, intracellular localization, signal transduction, stress responses, cell cycle progression and protein stability. Sumoylation depends on a SUMO activating enzyme (E1), a SUMO conjugating enzyme (E2), and SUMO ligases (E3) that facilitate sumoylation. In addition, specific SUMO proteases such as Ulps are required for processing of SUMO to its major form (C-terminal di-glycine motif). SUMO-conjugated proteins can be deconjugated by SUMO proteases (desumoylases) and released SUMO can be recycled (Gareau and Lima, 2010).

Remarkably, bacterial effectors may possess SUMO protease activity. The T3 effector XopD of the plant pathogen *X. campestris* is a prototype of such a protease. XopD is a modular protein with a C-terminal SUMO protease domain that can process various plant SUMO isoforms (Hotson et al., 2003; Chosed et al., 2007; Kim et al., 2011). Moreover, XopD possesses deubiquitinase activity that depends on an unstructured ubiquitin-binding region, indicating a multi-functional enzyme (Pruneda et al., 2016). Proteolytic activity of XopD requires a catalytic triad (HDC residues) in the C-terminal SUMO protease (C48 cysteine peptidase) domain. In addition, DNA binding activity has been reported for XopD (Kim et al., 2008). Known plant target proteins of XopD proteins in *Arabidopsis thaliana* are transcription factors such as HFR1 (positive regulator of photomorphogenesis) (Tan et al., 2015) as well as DELLA proteins (negative regulators of gibberellin signaling) (Tan et al., 2014). In tomato (*Solanum lycopersicum*), XopD desumoylates the ethylene responsive transcription factor SIERF4 (Kim et al., 2013). Fluorescence-tagged XopD proteins expressed in plant cells are localized in nuclei and often accumulate in nuclear bodies that are referred to as nuclear foci in previous studies (Hotson et al., 2003). Nuclear bodies are distinct punctate structures in nuclei such as Cajal bodies and nuclear speckles (Morimoto and Boerkoel, 2013).

Several T3 effectors (or effector candidates) of rhizobia show certain sequence similarities with the C-terminal protease domain of XopD. The nodulation outer protein NopD (SFHH103_04358; CEO91485.1) of *Sinorhizobium fredii* HH103 was identified by mass spectrometry by comparing extracellular protein profiles from a T3SS-knockout mutant with the parent strain (Rodrigues et al., 2007). *Mesorhizobium loti* MAFF303099 secretes a related protein (mlr6316) in a T3SS-dependent manner. Mutant analysis and inoculation experiments with host plants suggested a possible symbiotic role of this protein in nodulation or nodulation competitiveness (Hubber et al., 2004; Sánchez et al., 2012). Moreover, the two recently identified bradyrhizobial proteins inducing *Rj4*-mediated nodulation blockage (BEL2_5, Faruque et al., 2015; MA20_12780, Tsurumaru et al., 2015) can be considered as NopD family proteins. On the molecular level, however, NopD proteins have not been studied yet.

In this work, we have characterized NopD of *Bradyrhizobium* sp. XS1150. NopD can process specific plant SUMO proteins and desumoylate SUMO-conjugated proteins. Moreover, we provide evidence that NopD expressed *in planta* is targeted to nuclei where it accumulates in nuclear bodies. NopD activity induces ETI-like plant responses, namely cell death in tobacco and reduced nodule formation on roots of the legume *Tephrosia vogelii*.

## Materials and Methods

### Strains, Plasmids and Primers

Information on strains and plasmids used in this study is provided in Supplementary Table S1. Plasmids were constructed according to standard methods with restriction enzymes and PCR-based methods. Primers are listed in Supplementary Table S2.

### Identification of a T3SS Gene Cluster and a *nopD* Gene in *Bradyrhizobium* sp. XS1150

*Bradyrhizobium* sp. XS1150 was isolated from a nodule of a peanut plant (*Arachis hypogaea* cv. Liaoning Silihong) at a suburban field close to Guangzhou, China (23.38920N, 113.39900E). Strain XS1150 is resistant to 10 μg/mL chloramphenicol and efficiently grows in various media (Supplementary Text 1). Genomic DNA of strain XS1150 was shotgun-sequenced by the company Ai Jian Genomics (Guangzhou, China) using the Illumina GA*_IIx_* system (Illumina). Genes on scaffolds were predicted by the Prodigal v2_60 software. Database comparisons were performed using the Basic Local Alignment Search Tool (BLAST) at the NCBI homepage^1^. The draft genome sequence of strain XS1150 has been deposited at DDBJ/ENA/GenBank (whole genome shotgun sequencing project NFUH00000000.1; Bioproject PRJNA385724). Using putative rhizobial T3SS genes and predicted T3 effectors (Staehelin and Krishnan, 2015) as query sequences, a T3SS gene cluster (in scaffold 201) and a *nopD* gene (in scaffold 90) were identified in XS1150. The coding sequence of *nopD* was PCR-cloned and confirmed by Sanger sequencing (accession number MF100854). Amino acid sequence alignment of the C-terminal part of NopD with related rhizobial proteins and the *Xanthomonas* effector XopD was performed with DNAstar. Lasergene.v7. A corresponding phylogentic tree was constructed with MEGA5 software using the neighbor-joining method and default setting. Bootstrap analysis was performed with 1000 replications (Tamura et al., 2011).

### Functional Analysis of the NopD Secretion Signal Sequence

To analyze functionality of the N-terminal secretion signal of NopD, the N-terminal secretion signal sequence (residues 1–50) of the effector NopL produced by *Sinorhizobium* sp. NGR234 was replaced by the corresponding N-terminal sequence of NopD. A DNA fragment consisting of the *nopL* promoter from NGR234, the *nopD* sequence (encoding amino acid residues 1–50) fused to *nopL* (encoding amino acid residues 51–338) was cloned into the RK2-derived cloning vector pFAJ1703 (Dombrecht et al., 2001). The plasmid, named pFAJ-NopD:NopL, was then mobilized into NGRΩ*nopL* (NGR234 derivative with an Ω interposon in the *nopL* gene; Marie et al., 2003) and NGRΩ*rhcN* (Ω interposon in the *rhcN* gene and thus lacking a functional T3SS; Viprey et al., 1998). Bacterial cultures (180 rpm, 27°C) were treated with 1 μM apigenin and harvested 45 h later. Proteins from culture supernatants were precipitated with 10% (w/v) trichloroacetic acid and used for SDS-PAGE, Ponceau staining and Western blot analysis with a previously prepared antibody against NopL (Zhang et al., 2011). Details are described in Supplementary Text 1.

### Recombinant Proteins Expressed in *Escherichia coli*

*Escherichia coli* BL21 (DE3) cells carrying a given plasmid were used for expression of recombinant proteins. Purification of proteins was carried out according to the manufacturer’s protocol for affinity chromatography of native proteins (for 6 × His-tagged proteins: Ni-NTA magnetic agarose beads from Qiagen, Germantown, MA, United States; for GST fusion proteins: glutathione agarose beads from Novagen, Madison, WI, United States). Purified proteins were subjected to SDS-PAGE, Western blot analysis or enzyme tests.

### SDS-PAGE and Western Blot Analysis

Proteins were separated by SDS-PAGE on 12% polyacrylamide gels and stained with Coomassie Brilliant Blue G-250. For Western blot analysis, proteins were separated onto nitrocellulose membranes. Membranes were incubated with commercially available antibodies against protein tags, with an antibody recognizing NopL of *Sinorhizobium* sp. NGR234 (Zhang et al., 2011) or against an antibody recognizing a C-terminal part of NopD of strain *Bradyrhizobium* sp. XS1150. For preparation of the anti-NopD antibody, recombinant NopD (residues 640–1017) with an N-terminal 6 × His tag was expressed in *E. coli* BL21 (DE3) and the purified protein was used for immunization of a rabbit. After incubation with horseradish peroxidase-conjugated second antibodies, Western blots were developed with 3,3′-diaminobenzidine (Boster, Wuhan, China) or by electrochemiluminescence detection reagents (Amersham GE Healthcare, Little Chalfont, United Kingdom) according to the supplier’s protocols.

### Peptidase and Isopeptidase Activity Assays

For the *in vitro* peptidase assay, purified substrates (GST fused to various SUMO-Gly-Gly-3HA) were incubated with purified 6 × His-tagged test proteins in elution buffer used for purification of 6 × His-tagged proteins (20 mM Tris–HCl, pH 7.9, containing 500 mM imidazole and 0.5 M NaCl) for 30 min at 30°C. Enzyme assays were performed with: (i) full-length NopD; (ii) NopD-C, the C-terminal domain of NopD (residues 640–1017 with an N-terminal methionine); and (iii) NopD-C_972_A, a NopD variant with a cysteine-to-alanine substitution in the catalytic core. Reaction mixtures were then analyzed on Western blots with an anti-GST antibody. Removal of the C-terminal 3HA tag resulted in a clear band shift.

For the isopeptidase assay, a commonly used *in vitro* desumoylation assay was performed with sumoylated RanGAP of *Homo sapiens* (Matunis et al., 1996). To prepare the substrates, different SUMO proteins (processed forms with terminal Thr-Gly-Gly residues) were conjugated to the acceptor RanGAP (with an N-terminal 6 × His tag and a C-terminal Myc tag) by using recombinant E1 and E2 proteins of *A. thaliana*. Purified 6 × His-tagged AtSAE1 (E1), AtSAE2 (E1) and AtUbc9 (E2) were prepared for this purpose. The sumoylation reaction was carried out in a total volume of 100 μl with 8 μg of RanGAP-Myc-6 × His, 8 μg of GST-SUMO(TGG), 1 μg of AtSAE1-His_6_, 1 μg of AtSAE2-His_6_ and 2 μg of AtUbc9-His_6_ in 50 mM Tris–HCl buffer (pH 7.8) containing 100 mM NaCl, 15% glycerol, 5 mM ATP, and 10 mM MgCl_2_ at 22°C for 6 or 8 h. No sumoylated RanGAP was formed when the SUMO protease XopD of *X. campestris* (also expressed as 6 × His tagged protein) was added to the reaction mixture. The reaction products containing RanGAP conjugated to different SUMO proteins were then incubated (30°C; 30 min) with 0.1 μg of 6 × His-tagged enzymes (NopD, NopD-C and NopD-C_972_A). Removal of SUMO from sumoylated RanGAP-Myc-His_6_ forms was analyzed on Western blots with an anti-Myc antibody.

### Expression of NopD and Variants in Plant Cells

*Agrobacterium*-mediated transient gene expression in tobacco (*N. tabacum* cv. Xanthi) was used for expression of NopD and enzymatically inactive NopD-C_972_A (cysteine-to-alanine substitution in the catalytic core). In subcellular localization studies, NopD fused to YFP were expressed in *A. thaliana* protoplasts. In a similar way, NopD variants fused to YFP were analyzed, namely (i) enzymatically inactive NopD-C_972_A, (ii) NopD-N, the N-terminal domain of NopD (residues 1–390), (iii) NopD-NΔ2-53, a NopD-N variant lacking residues 2–53, (iv) NopD-NΔ2-60, a NopD-N variant lacking residues 2–60; (v) NopD-TR, the tandem repeat domain of NopD (residues 391–720 with an N-terminal methionine), and (vi) NopD-C, the C-terminal protease domain of NopD (residues 640–1017 with an N-terminal methionine). ARF4 (auxin response factor 4 of *A. thaliana*) fused to RFP served as nuclear marker. Details on protein expression in tobacco and *A. thaliana* are shown in Supplementary Text 1.

### Construction of XS1150 Mutants

The mutant XS1150Ω*rhcST* (lacking a functional T3SS) was constructed by inserting an ΩSpe interposon into the T3SS apparatus gene *rhcS* of strain *Bradyrhizobium* sp. XS1150. Strain XS1150Δ*nopD, a nopD*-deficient mutant of XS1150, was constructed by replacing the *nopD* coding sequence with an ΩSpe interposon. Strain XS1150Δ*nopD*+*nopD* is a derivative of XS1150Δ*nopD* in which the *nopD* gene (including a 1-kb promoter region) was re-introduced. The mutant XS1150Δ*nopD*+*nopD*-C_972_A was constructed in a similar way to obtain a strain that produces an enzymatically inactive NopD variant (substitution of cysteine residue 927 by alanine). Details on the mutant construction procedure are provided in Supplementary Text 1 and Supplementary Figure S1).

### Nodulation Tests

*Tephrosia vogelii* was used to characterize the symbiotic phenotypes of the constructed mutants (XS1150Δ*nopD*, XS1150Δ*nopD*+*nopD*, XS1150Δ*nopD*+*nopD*-C_972_A and XS1150Ω*rhcST*) as compared to the parent strain *Bradyrhizobium* sp. XS1150. Information on performed nodulation tests can be found in Supplementary Text S1. Statistical analysis was performed by Kruskal–Wallis tests considering each plastic jar unit (1 plant) as a replicate.

### Accession Numbers

Sequences used for DNA constructs of this study have the following accession numbers in sequence databases: Draft genome of *Bradyrhizobium* sp. (*B. guangdongense*) XS1150: NFUH00000000 (BioProject PRJNA385724); NopD of *Bradyrhizobium* sp. XS1150: MF100854; NopL of *Sinorhizobium* sp. NGR234: NC_000914; AtSUMO1 of *A. thaliana*: AEE85259; AtSUMO2 of *A. thaliana*: NM_124898; AtSUMO3 of *A. thaliana*: NM_124899; AtSUMO5 of *A. thaliana*: NM_128836; HuSUMO1 of *H. sapiens*: AK311840; HuSUMO2 of *H. sapiens*: AK311837; HuSUMO4 of *H. sapiens*: AB205057; PvSUMO of *Phaseolus vulgaris*: XM_007146455; GmSUMO of *G. max*: NM_001248279; Smt3 of *Saccharomyces cerevisiae*: CP020194; AtSAE1 of *A. thaliana*: BT000094; AtUbc9 of *A. thaliana*: NM_001202641; AtSAE2 of *A. thaliana*: BT003377; RanGAP of *H. sapiens*: NM_001317930.1; ARF4 of *A. thaliana*: NP_200853.

## Results

### Identification of *nopD* in the Genome of *Bradyrhizobium* sp. XS1150

*Bradyrhizobium* sp. XS1150 was isolated from a peanut (*A. hypogaea*) nodule at a suburban field close to Guangzhou, China. Re-inoculation tests resulted in efficient nodule formation that promoted growth of peanuts. Strain XS1150 induced also nodules on roots of *T. vogelii* (Supplementary Figure S2). Whole-genome shotgun sequencing revealed that XS1150 is a *Bradyrhizobium* strain (tentatively named *Bradyrhizobium guangdongense*). The sequences (670 contigs; totally 7624764 nucleotides) were submitted to the DDBJ/ENA/GenBank database (accession number NFUH00000000). Sequence homology searches indicated that the XS1150 genome possesses a T3SS gene cluster that contains the transcriptional regulator gene *ttsI* and the putative effector genes *nopL*, *nopE1*, and *nopP*. Moreover, a sequence homologous to the effector gene *nopAR* of *B. japonicum* USDA122 (=bll1840 in strain USDA110; Tsukui et al., 2013) was found in the T3SS gene cluster of XS1150 (Supplementary Figure S3).

**FIGURE 1:**
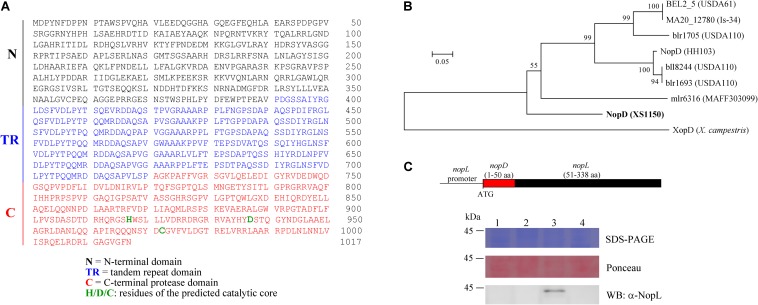
NopD of *Bradyrhizobium* sp. XS1150. **(A)** Amino acid sequence of NopD, a modular protein that consists of an N-terminal domain (N), a tandem repeat domain (TR) and a C-terminal protease domain (C) with histidine, aspartic acid and cysteine residues (predicted catalytic triad). **(B)** Phylogenetic analysis of NopD family proteins based on a conserved C-terminal region. The residues 835–982 of NopD were aligned with a selection of related rhizobial proteins (*B. elkanii* USDA61, *B. japonicum* Is-34, *B. japonicum* USDA110, *M. loti* MAFF303099 and *Sinorhizobium fredii* HH103) and with XopD of *X. campestris* pv. *campestris* 8004. The alignment and accession numbers of the proteins are shown in Supplementary Figure S4. The tree was constructed with MEGA5 software. Bootstrap values are indicated next to branches. The scale bar represents 0.05 substitutions per site. **(C)** T3SS-dependent secretion of a chimeric NopD-NopL protein. The DNA construct of plasmid pFAJ-NopD:NopL is shown on the top of the panel. The plasmid was mobilized into the NGRΩ*nopL* and NGRΩ*rhcN* mutants of *Sinorhizobium* sp. NGR234. Equal amounts of secreted proteins from culture supernatants were analyzed by SDS-PAGE and Ponceau staining (loading control of Western blot). NopD-NopL was immunodetected by an antibody against NopL. Lane 1, NGRΩ*nopL*; lane 2, NGRΩ*rhcN*; lane 3: NGRΩ*nopL* carrying pFAJ-NopD:NopL; lane 4, NGRΩ*rhcN* carrying pFAJ-NopD:NopL.

Using the C-terminal protease domain sequence of *nopD* from *Sinorhizobium fredii* HH103 (accession number CEO91485.1) as query sequence, an additional putative effector gene of XS1150 was identified outside the T3SS gene cluster of XS1150. The NopD protein of strain XS1150 possesses a calculated molecular weight of 111.45 kDa. It consists of an N-terminal domain (residues 1–390), a tandem repeat (TR) domain with 7 repeats (residues 391–720; the first 6 repeats contain 49 residues, the last one contains 36 residues) and a C-terminal protease domain (residues 721–1017) (Figure 1A). The C-terminal protease domain of NopD shows sequence similarities to NopD of strain HH103 and to various other putative rhizobial effectors (such as BEL2_5 of USDA61, MA20_12780 of Is-34, mlr6316 of MAFF303099, bll8244 of USDA110, blr1693 of USDA110, blr1705 of USDA110). The C-terminal protease domains of these rhizobial proteins could be aligned to the *Xanthomonas* effector XopD, a SUMO protease of the C48 cysteine peptidase family (Supplementary Figure S4). The alignment allowed prediction of conserved residues (catalyctic triad) required for SUMO protease activity. Based on the obtained alignment, a corresponding phylogenetic tree was constructed (Figure 1B).

### NopD Possesses a Functional Secretion Signal Sequence

Bioinformatic analysis with EffectiveDB (Eichinger et al., 2016) predicted that NopD possesses an N-terminal secretion signal sequence required for T3SS-dependent secretion. To confirm this prediction, we prepared a plasmid (named pFAJ-NopD:NopL) to express a chimeric NopD-NopL protein in the mutants NGRΩ*nopL* and NGRΩ*rhcN* of *Sinorhizobium* sp. NGR234. NGRΩ*nopL* is a knockout mutant deficient in synthesis of the NopL effector and NGRΩ*rhcN* lacks a functional T3SS. Figure 1C shows a schematic view of the expressed construct. Western blot analysis with an anti-NopL antibody indicated presence of the NopD-NopL protein in the culture supernatant of NGRΩ*nopL* carrying pFAJ-NopD:NopL. However, no corresponding Western blot signals were observed for protein preparations from the culture supernatant of NGRΩ*rhcN* carrying pFAJ-NopD:NopL (Figure 1C). These findings indicate that NopD possesses an N-terminal secretion signal sequence that is recognized by the T3SS of strain NGRΩ*nopL*.

### NopD Is a SUMO Protease

A truncated NopD protein (residues 640–1017) was expressed in *E. coli* in order to produce a polyclonal antibody against NopD. The protein with a 6 × His-tag was purified by nickel affinity purification and then used for immunization of a rabbit. Full-length His-tagged NopD, albeit to a lesser extent, could also be expressed in *E. coli.* After purification by nickel affinity chromatography, a band corresponding to the expected molecular weight was detected with the prepared antibody. In addition, faster migrating bands (presumably degraded NopD forms) were observed (Supplementary Figure S5).

**FIGURE 2:**
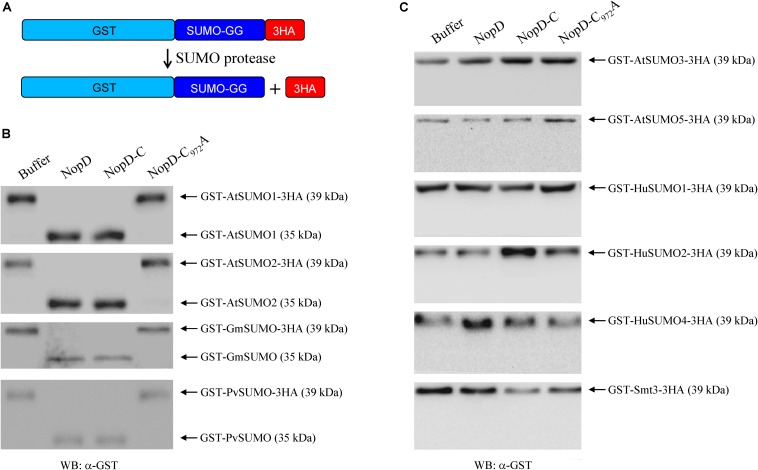
Peptidase activity of 6 × His-tagged NopD and NopD-C. **(A)** Schematic view of the test system that is based on release of the C-terminal 3HA tag from a given GST-SUMO protein by NopD and NopD-C (C-terminal protease domain of NopD). Reaction mixtures were incubated at 30°C for 1 h. 6 × His-tagged NopD-C_972_A (enzymatically inactive variant) and buffer (no enzyme) were used as negative controls. **(B)** Analysis of reaction mixtures on Western blots probed with an anti-GST antibody. Incubation of GST-AtSUMO1-3HA, GST-AtSUMO2-3HA, GST-GmSUMO1-3HA and GST-PvSUMO1-3HA with 6 × His-tagged NopD or NopD-C resulted in band shifts, indicating release of the C-terminal 3HA tag. Incubation with NopD-C_972_A or buffer alone did not result in a band shift. **(C)** Results for reactions with GST-SUMO-3HA proteins that were not cleaved by NopD or variants (no obvious band shift).

As the C-terminal protease domain of NopD proteins is related to the *Xanthomonas* T3 effector XopD, we expected that NopD possesses SUMO protease activity. We expressed full-length NopD, NopD-C_972_A (substitution of the predicted catalytic core cysteine residue to alanine) and NopD-C (C-terminal protease domain) in *E. coli* as 6 × His-tagged proteins. SUMO proteins with a GST tag at the N- terminus and three HA tags at the C-terminus (directly following the C-terminal Gly-Gly residues) were also expressed in *E. coli*. Such GST-SUMO1-3HA fusion proteins were prepared for various SUMOs from *A. thaliana* (AtSUMO1, AtSUMO2, AtSUMO3 and AtSUMO5), soybean (GmSUMO), common bean (PvSUMO), human (HuSUMO1, HuSUMO2 and HuSUMO4) and yeast (*S. cerevisiae*; Smt3). The native proteins, purified by affinity chromatography, were then used for hydrolytic tests. As shown in Figure 2, full-length NopD and NopD-C, but not NopD-C_972_A, had the capacity to release the three HA tag from GST-AtSUMO1-3HA (*A. thaliana*), GST-AtSUMO2-3HA (*A. thaliana*), GST-GmSUMO-3HA (soybean) and GST-PvSUMO-3HA (common bean). However, the two other *Arabidopsis* SUMO isoforms (GST-AtSUMO3-3HA and GST-AtSUMO5-3HA) were not cleaved in this assay. Likewise, SUMO isoforms from human (GST-HuSUMO1-3HA, GST-HuSUMO2-3HA, GST-HuSUMO4-3HA) as well as GST-Smt3-3HA from yeast were no substrates for NopD or NopD-C. Hence, NopD and NopD-C could process the C-terminal end of specific plant SUMO proteins.

**FIGURE 3:**
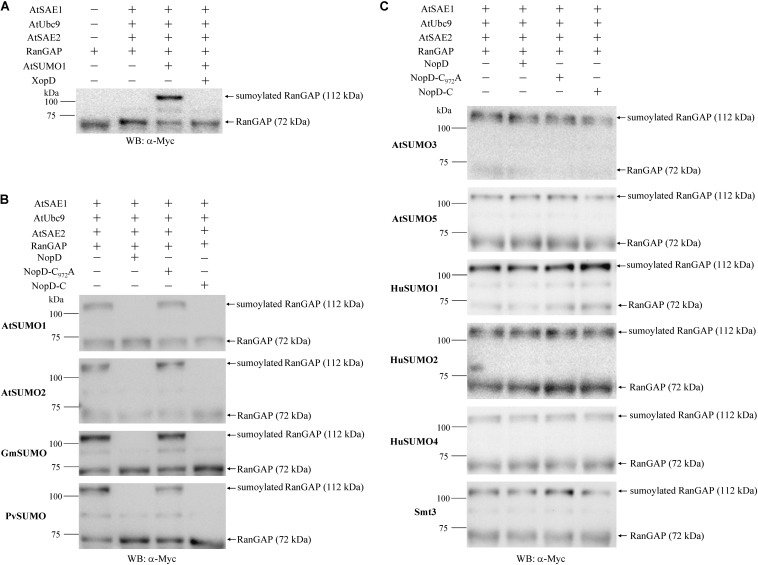
Isopeptidase activity of 6 × His-tagged NopD and NopD-C. SUMO-RanGAP conjugates were prepared to analyze the desumoylation activity of NopD and NopD-C. **(A)** Preparation of an AtSUMO1-RanGAP conjugate in an *in vitro* sumoylation system with indicated 6 × His-tagged *Arabidopsis* proteins and human RanGAP (with 6 × His and Myc tags). The obtained AtSUMO1-RanGAP conjugate was not formed in the presence of 6 × His-tagged XopD. Western blot analysis of sumoylated RanGAP and RanGAP was performed with an anti-Myc antibody. Similar SUMO-RanGAP conjugates were obtained for other SUMO proteins (see Supplementary Figure S6). **(B)** NopD and NopD-C show SUMO isopeptidase activity for indicated SUMO-RanGAP conjugates. Incubation with 6 × His tagged NopD and NopD-C resulted in desumoylation of the conjugate, whereas NopD-C_972_A (enzymatically inactive) or buffer (no enzyme) showed no effects. Reaction mixtures were analyzed on Western blots probed with an anti-Myc antibody. **(C)** Western blot results of reactions with indicated SUMO-RanGAP conjugates that were not desumoylated by NopD or NopD-C under the same test conditions.

SUMO proteases not only process SUMO proteins but can also remove SUMO from SUMO conjugated acceptor proteins. To investigate whether NopD has such isopeptidase activity, we cloned *Arabidopsis* genes of the sumoylation cascade, namely *AtSAE1*, *AtSAE2* and *AtUbc9*. These genes were subsequently expressed in *E. coli* as 6 × His-tagged proteins and purified. Similarly, we prepared the acceptor protein RanGAP of *H. sapiens* (with an N-terminal 6 × His tag and a C-terminal Myc tag) and AtSUMO1 in its processed form (TGG), fused to an N-terminal GST tag. The recombinant proteins were used to obtain sumoylated RanGAP. Formation of an AtSUMO1-RanGAP conjugate was not observed when the known SUMO protease XopD was added to the reaction (Figure 3A). Other SUMO-RanGAP conjugates were prepared in a similar way (Supplementary Figure S6). The proteins were then used for isopeptidase activity tests with NopD and variants. Reactions with NopD and NopD-C resulted in desumoylation of AtSUMO1-RanGAP, AtSUMO2-RanGAP, GmSUMO-RanGAP or PvSUMO-RanGAP. In contrast, NopD-C_972_A did not show enzyme activity (Figure 3B). All other conjugates (AtSUMO3-RanGAP, AtSUMO5-RanGAP, HuSUMO1-RanGAP, HuSUMO2-RanGAP, HuSUMO4-RanGAP and Smt3-RanGAP) remained intact when incubated with NopD or NopD-C (Figure 3C). Hence, isopeptidase activities of NopD and NopD-C were similar to those obtained with GST-SUMO-3HA proteins.

### NopD but Not NopD-C_972_A Induces Cell Death in Tobacco

**FIGURE 4:**
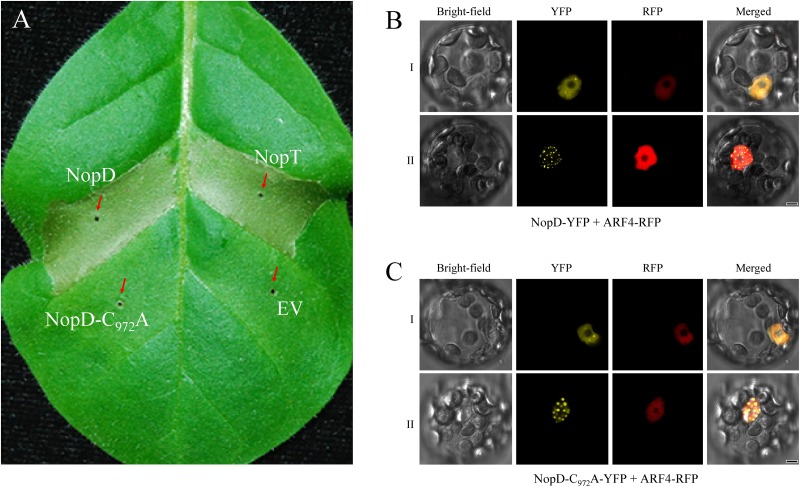
Expression of NopD and NopD-C_972_A in plant cells. **(A)** NopD induces cell death in tobacco. Expression of NopD and NopD-C_972_A in different sections of a tobacco leaf was performed by infiltration of *A. tumefaciens* carrying appropriate plasmids (pCAMBIA1302 derivatives). NopT (a T3 effector of *Sinorhizobium* sp. NGR234) was used for comparison. The photograph was taken 48 h after bacterial infiltration. Cell death (necrotic tissue) was observed for NopD and NopT, whereas expression of NopD-C_972_A showed no visible effects. **(B,C)** Subcellular localization of YFP-tagged NopD and NopD-C_972_A in *Arabidopsis* protoplasts. The nuclear marker ARF4 fused to RFP was co-expressed. Cells were analyzed with a confocal microscope for red fluorescence (RFP) emission, yellow fluorescence (YFP) emission and under bright-field illumination (18 h after transformation). YFP fluorescence was evenly distributed throughout the nucleus (type I cells) or preferentially in nuclear bodies (type II cells).

To study effects of NopD in living plant cells, we transiently expressed NopD in tobacco cells. *Agrobacterium tumefaciens* carrying binary vectors containing the CaMV 35S promoter and a given *nopD* sequence were infiltrated into leaves of tobacco plants. Remarkably, a rapid cell death response was induced by expression of NopD. The strength of the hypersensitive response was comparable to that induced by the effector NopT of *Sinorhizobium* sp. NGR234 (Dai et al., 2008). However, NopD-C_972_A expression in tobacco did not cause cell death (Figure 4A). These data indicate that the observed hypersensitive response depended on the protease activity of NopD.

### Subcellular Localization of NopD and Variants in Plant Nuclei

To investigate the subcellular localization of NopD in plant cells, NopD variants fused to YFP were expressed in *Arabidopsis* protoplasts. The constructs were expressed from the CaMV 35S promoter. Analysis of transformed protoplasts by confocal microscopy revealed that fluorescence of NopD-YFP appeared in nuclei although no classic nuclear localization signal was found in NopD (Figure 4B). The ARF4 protein of *A. thaliana* fused to RFP was used as nuclear marker. We noticed that the distribution of NopD-YFP in the nucleus was of two types: (i) fluorescence distributed evenly throughout the nucleus and (ii) fluorescence predominantly localized to nuclear bodies as reported previously for the effector *Xanthomonas* effector XopD (Hotson et al., 2003). Over time, the strength of fluorescence signals increased in the nuclear bodies, suggesting that NopD-YFP was first homogeneously localized in the nucleus and then re-localized to the nuclear bodies. The enzymatically inactive variant NopD-C_972_A fused to YFP showed a similar nuclear localization pattern (Figure 4C). In contrast, NopD-N (residues 1–390) fused to YFP did not accumulate in nuclear bodies but was localized in the nucleus (with strong fluorescence signals in the nucleolus), suggesting the presence of a cryptic nuclear localization signal in the N-terminal domain of NopD. Removal of N-terminal residues from NopD-N (T3SS secretion signal sequence) had no impact, i.e. localization of YFP-tagged NopD-N lacking residues 2–53 (NopD-NΔ2-53) or 2–60 (NopD-NΔ2-60) was not different from NopD-N. YFP-tagged NopD-TR (tandem repeat domain of NopD; residues 391–720) and NopD-C (C-terminal SUMO protease domain; residues 640–1017) were evenly distributed in the cell like YFP alone (Supplementary Figure S7).

### NopD Negatively Affects Nodulation of the Host Plant *T. vogelii*

**FIGURE 5:**
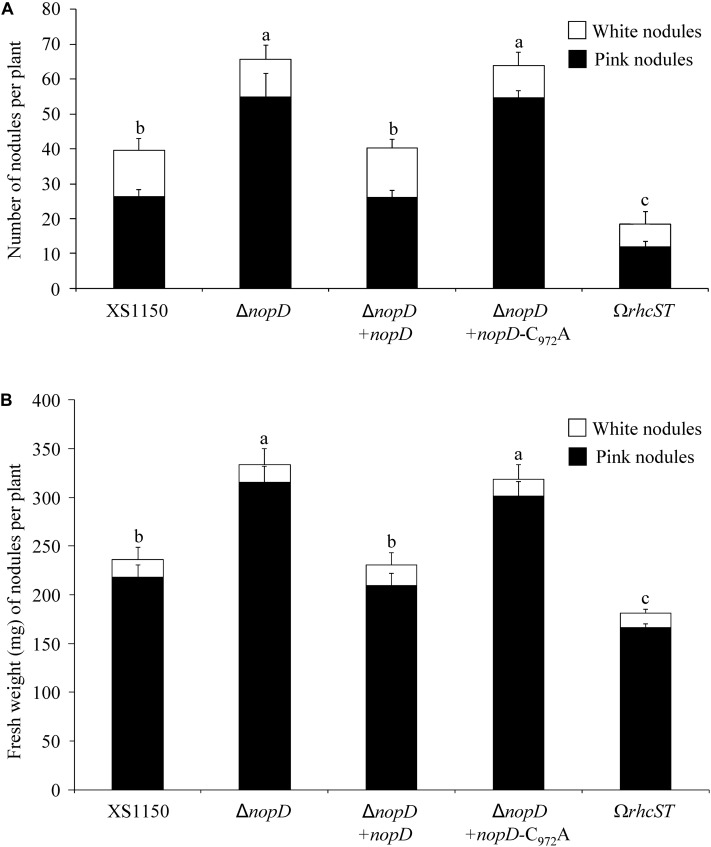
Symbiotic phenotype of *Bradyrhizobium* sp. XS1150 and constructed mutants on the host plant *T. vogelii*. Data shown are the results of a representative nodulation test. Plants were inoculated with indicated strains and harvested 36 days later. Data indicate means ± SE (8 jars; *n* = 8). Different letters indicate significant differences (Kruskal–Wallis tests, *P* < 0.02). **(A)** Nodule number (number of pink nodules and white nodules per plant). **(B)** Nodule biomass (fresh weight of pink and white nodules per plant). Abbreviations: XS1150, *Bradyrhizobium* sp. XS1150 (wild-type); Δ*nopD*, XS1150Δ*nopD* (*nopD* knockout mutant); Δ*nopD*+*nopD*, XS1150Δ*nopD*+*nopD* (rescued *nopD* knockout mutant); Δ*nopD*+*nopD*-C_972_A, XS1150Δ*nopD*+*nopD*-C_972_A (*nopD* knockout mutant expressing NopD-C_972_A); Ω*rhcST*, XS1150Ω*rhcST* (mutant lacking a functional T3SS).

To explore symbiotic effects of NopD during symbiosis, a *nopD* deletion mutant of *Bradyrhizobium* sp. XS1150, named XS1150Δ*nopD*, was constructed (Supplementary Figure S1). Nodulation tests with various legumes revealed that *T. vogelii* is a host plant that differently responds to XS1150 and XS1150Δ*nopD*. The mutant induced significantly more nodules and the nodule biomass per plant was also increased. These findings suggest that NopD functions as asymbiotic effector that negatively affects the symbiosis between strain XS1150 and *T. vogelii*. Compared to the parent strain XS1150, less nodules and a lower nodule biomass per plant were observed when *T. vogelii* plants were inoculated with the T3SS knockout mutant XS1150Ω*rhcST.* These findings suggest that uncharacterized effectors of XS1150 show symbiosis-promoting effects (Figure 5).

Re-introduction of *nopD* into the XS1150Δ*nopD* mutant resulted in a *nopD* expressing strain (XS1150Δ*nopD*+*nopD*). Moreover, we introduced a modified *nopD* sequence (C_972_A substitution) into XS1150Δ*nopD* to create a mutant that produces enzymatically inactive NopD-C_972_A and named the strain XS1150Δ*nopD*+*nopD*-C_972_A (Supplementary Figure S1). As expected, nodulation of XS1150Δ*nopD*+*nopD* on *T. vogelii* roots resulted in nodulation parameters comparable to strain XS1150, indicating that the wild-type phenotype was restored. In contrast, the symbiotic phenotype of strain XS1150Δ*nopD*+*nopD*-C_972_A was not different from XS1150Δ*nopD* (Figure 5). Hence, the cysteine residue 972 required for SUMO protease activity was indispensable for the NopD effect in the interaction with *T. vogelii.*

## Discussion

In this study, we have characterized a putative T3 effector of *Bradyrhizobium* sp. XS1150. NopD and the C-terminal domain alone (NopD-C) show SUMO processing activity and SUMO deconjugation activity. The cysteine residue 972 of NopD was found to be essential for enzyme activity in these tests. We propose to use the protein name NopD for all rhizobial T3 effectors with an enzymatically active SUMO protease domain (C48 or Ulp1 peptidase family) even if other domains in these effectors are different or absent.

The NopD-NopL fusion protein was secreted by the T3SS of strain NGRΩ*nopL* whereas no Western blot signal was observed for the T3SS-deficient mutant NGRΩ*rhcN.* These findings indicate that the N-terminal sequence of NopD is a functional secretion signal sequence as predicted by EffectiveDB (Eichinger et al., 2016). T3SS-dependent NopD secretion by XS1150 and translocation into legume cells remains to be experimentally confirmed. Support for translocation into host cells is provided by our findings that NopD can target plant SUMO proteins and that nodulation of *T. vogelii* was negatively affected by the catalytic cysteine residue 972 in NopD. Remarkably, NopD and NopD-C (C-terminal protease domain) both showed a rigid substrate preference for specific plant SUMO proteins, i.e. they can process only specific GST-SUMO-3HA proteins (AtSUMO1, AtSUMO2, GmSUMO and PvSUMO). A similar preference for the same plant SUMO protein was observed when NopD was used in a SUMO deconjugation assay with sumoylated RanGAP. All SUMO proteins processed by NopD contain a C-terminal recognition motif previously identified for XopD (A_35_-R_29_-M_7_L_6_H_5_Q_4_T_3_G_2_G_1_; numbers following the amino acid residues indicate positions relative to the cleavage site; see Supplementary Figure S8; Chosed et al., 2007). Other SUMO proteins lacking this motif (particularly M_7_L_6_H_5_ residues) were not processed by NopD or XopD. Hence, NopD and XopD appear to possess a similar substrate preference for SUMOs and thus may target plant proteins that are sumoylated in a similar way. However, the N-terminal parts of NopD and XopD proteins are rather different (residues 1–720 of NopD show only 17% amino acid sequence identity with XopD of *X. campestris* pv. campestris strain 8004).

Although various rhizobial T3 effectors have been identified (Staehelin and Krishnan, 2015), subcellular localization analysis in plant cells has been only performed for few effectors of *Sinorhizobium* sp. NGR234 (Dowen et al., 2009; Ge et al., 2016; Xu et al., 2018) and *Bradyrhizobium* sp. ORS3257 (Teulet et al., 2019). Fluorescence-tagged NopD was found to be targeted to the plant nucleus (Figure 4) although the protein apparently lacks a classic nuclear localization signal. We suggest that NopD possesses a cryptic nuclear localization signal in its N-terminus as YFP-tagged NopD-N (residues 1–390) also showed nuclear localization. NopD and NopD-C_972_A (Figure 4), but not other NopD variants (Supplementary Figure S7), accumulated in nuclear bodies. Hence, subnuclear localization depended on full-length NopD whereas protease activity was not required for accumulation of NopD in nuclear bodies. The *Xanthomonas* T3 effector XopD expressed in plant cells may also accumulate in nuclear bodies (Hotson et al., 2003). Co-expression of XopD proteins with a given target protein (SlERF4 or HFR1) resulted in co-localization of both proteins in nuclear bodies (Kim et al., 2013; Tan et al., 2015).

Like T3 effectors from pathogens, rhizobial effectors are expected to suppress plant defense reactions, thereby promoting rhizobial infection, nodule formation and survival of bacteroids in nodules (Staehelin and Krishnan, 2015; Nelson and Sadowsky, 2015; Cao et al., 2017). On the other hand, rhizobial effectors can have negative effects on symbiosis with certain legumes. The role of NopD in the interaction between strain XS1150 and *T. vogelii* suggests that the protein is an asymbiotic effector similar to ETI-inducing avirulence proteins in plant-pathogen interactions. Likewise, the hypersensitive reaction of tobacco cells elicited by NopD expression can be considered as an ETI response. The proteolytically inactive NopD-C_972_A variant did not elicit cell death, however. This finding suggests indirect effector recognition through desumoylation of a NopD substrate that perhaps functions as sensor for disease resistance protein-mediated ETI (Cui et al., 2015). Nodulation tests with *T. vogelii* showed that the symbiotic effector activity of NopD also depended on cysteine residue 972. This finding suggests that abnormal desumoylation events caused by proteolytically active NopD were not favorable for nodulation of this plant. The NopD-dependent nodulation phenotype of *T. vogelii* is reminiscent of the incompatible interaction between *Rj4/Rj4* soybeans and *B. elkanii* USDA61 and *B. japonicum* Is-34. The C-terminal regions of the BEL2_5 (USDA61) and MA20_12780 (Is-34) proteins are related to NopD (Figure 1B) and thus are predicted to possess SUMO protease activity. Mutant strains lacking these proteins gained the ability to induce nodules on *Rj4/Rj4* soybeans, suggesting that NopD family proteins possess asymbiotic effector activity on soybean genotypes expressing *Rj4* (Faruque et al., 2015; Tsurumaru et al., 2015; Yasuda et al., 2016). *Rj4* encodes a specific thaumatin-like protein that only differs in few amino acids from homologs (Tang et al., 2016).

Taken together, we have identified and characterized NopD of *Bradyrhizobium* sp. XS1150. NopD is a modular protein that consists of at least three different units: (i) an N-terminal domain which appears to be required for T3SS-dependent secretion and that also contains information for NopD targeting into plant nuclei; (ii) a middle tandem repeat domain, and (iii) a C-terminal protease domain which targets specific plant SUMO proteins. The protease activity of NopD was required for cell death induction in tobacco and negatively affected nodule formation of *T. vogelii*. Future work will be required to identify SUMO-conjugated targets of NopD in *T. vogelii* or other host legumes.

## Data Availability Statement

The nucleotide sequences generated for this study can be found in the GenBank database (Bioproject PRJNA385724 and accession MF100854). Non-cropped images of SDS-PAGE gels and Western blots are shown in Supplementary Material.

## Author Contributions

Q-WX, Z-PX, and CS conceived and designed the experiments. Q-WX, JB, JC, Q-YH, YW, YL, and ZZ performed the experiments. Q-WX, JB, JC, Q-YH, YW, YL, ZZ, Z-PX, and CS analyzed the data. Q-WX, CW, Z-PX, and CS wrote the manuscript. All authors read and approved the manuscript.

## Conflict of Interest

The authors declare that the research was conducted in the absence of any commercial or financial relationships that could be construed as a potential conflict of interest.

## Footnotes

1 https://blast.ncbi.nlm.nih.gov/Blast.cgi

## Abbreviations

CaMV: cauliflower mosaic virus
E1: SUMO activating enzyme
E2: SUMO conjugating enzyme
E3: SUMO ligase
ETI: effector-triggered immunity
GST: glutathione S-transferase
PAMP: pathogen-associated molecular pattern
PTI: PAMP-triggered immunity
RFP: red fluorescence protein
SUMO: small ubiquitin-related modifier
T3: type III (effector)
T3SS: type III protein secretion system
Ulp: ubiquitin-like protein-specific protease
YFP: yellow fluorescence protein.
