# Supplementary material for: NopD of Bradyrhizobium sp. XS1150 Possesses SUMO Protease Activity
Source: Front Microbiol. 2020 Mar 20;11:386. doi: 10.3389/fmicb.2020.00386 (PMC7098955; doi:10.3389/fmicb.2020.00386)
Supplement: Supplementary file 1 [file Data_Sheet_1.PDF]

NopD of *Bradyrhizobium* sp. XS1150 possesses SUMO protease activity

Qi-Wang Xiang, Juan Bai, Jie Cai, Qin-Ying Huang, Yan Wang, Ying Liang, Zhi Zhong, Christian Wagner, Zhi-Ping Xie, and Christian Staehelin

Supplementary Text 1: Additional information on used materials and methods

Rhizobial media

Bradyrhizobium sp. XS1150 was found to efficiently grow in TY (Beringer 1974), arabinose-gluconate (Sadowsky et al. 1987), or YMA (Vincent 1970) media. *Sinorhizobium* sp. NGR234 mutants were grown in RMS medium using 12 mM succinate as carbon source (Broughton et al. 1986). All rhizobial strains were cultivated at 27 ºC.

Analysis of secreted proteins

Proteins secreted by *Sinorhizobium* sp. NGR234Ω*nopL*, NGR234Ω*rhcN*, NGRΩ*nopL* carrying pFAJ-NopD:NopL and NGRΩrhcN carrying pFAJ-NopD:NopL were prepared according to previously described procedures (Hempel et al., 2009; Marie et al., 2003). Bacteria were cultured in 500 ml RMS medium and expression of *nop* genes was induced with 1 µM apigenin (Sigma-Aldrich) when the cultures on the rotary shaker (180 rpm, 27 ºC) reached an OD_{600} of 0.05-0.1. Cultures were harvested 45 h after flavonoid induction. Cells were centrifuged (4 000 g, 4 ºC, 60 min) and the culture supernatants were centrifuged again (8 000 g, 4 ºC, 30 min) to remove all bacteria. Proteins in the culture supernatants were precipitated by adding solid trichloroacetic acid (TCA) to reach a final concentration of 10% (w/v). The TCA was dissolved by gently shaking the flasks. After incubation at 4 ºC for 24 h, samples were centrifuged (8000 g, 4 ºC, 45 min). The precipitates were washed twice with 2 ml of 80% ice-cold acetone and centrifuged again (6000 g, 4 ºC, 1 min). Finally, each precipitate was re-suspended in 100 µl rehydration buffer (8 M urea, 2% (w/v) 3-(3-cholamidopropyl)-dimethylammonio-1-propane sulfonate). Equal amounts of proteins were separated on 12% SDS-PAGE gels. Western blot analysis was performed with a previously prepared anti-NopL antibody using a 1:5000 dilution (Zhang et al. 2011). Membranes were stained with Ponceau S to confirm equal amounts of blotted proteins.

Expression of NopD and variants in plant cells

Agrobacterium-mediated transient gene expression in tobacco (*N. tabacum* cv. Xanthi) was performed according to Zhang and Liu (2001). The plants were kept in a greenhouse with a 16/8 h light/dark cycle. Leaves of 4- to 5-week-old plants were used for transformation. The binary vectors with a CaMV 35S promoter sequence and a given construct (encoding 6×His-tagged NopD or NopD-C_{972}A) were mobilized into *A. tumefaciens* strain EHA105 by electroporation. Bacterial suspensions (suspended in 10 mM $MgSO_4$ with 5 µM acetosyringone;

OD_{600} adjusted to 0.6 and incubated at 25 °C for 1 to 3 h) were infiltrated into leaves by the use of needleless syringes. Photographs were taken 48 h later. Extraction of proteins from infiltrated leaf tissue was performed as described (Bartsev et al. 2004). Western blot analysis with anti-NopD antibodies confirmed expression of NopD-C_{972}A in transformed tobacco cells. NopD expression was detected in staurosporine-treated leaves that showed reduced cell death symptoms.

Transient expression of fluorescence-tagged proteins (NopD and variants; ARF4) in *Arabidopsis* protoplasts was performed by using a transfection method with polyethylene glycol (PEG4000) (Yoo et al. 2007). All constructs contained the CaMV 35S promoter. Constructed plasmids (10 µg per sample) were used for transfection of mesophyll protoplasts (2 x 10^4 cells) prepared from *A. thaliana* ecotype Columbia. DNA for co-transfection experiments was combined prior to protoplast transfection. After transfection, protoplasts were incubated at room temperature under low light conditions. Localization of fluorescent proteins in protoplasts was analyzed by using a confocal fluorescence microscope (Leica, TCS SP5).

Construction of mutants of *Bradyrhizobium* sp. strain XS1150

Bradyrhizobium sp. XS1150Ω*rhcST* was constructed by insertion of an ΩSpe interposon (Prentki and Krisch 1984) into the *Hin*dIII site of the conserved T3SS apparatus gene *rhcS* of wild-type XS1150 (see Supplementary Figure S2). For mutant construction, the suicide vector pJQ-200SK (Quandt and Hynes 1993) and a 1.5-kb genomic DNA fragment containing the *rhcRST* genes of XS1150 were used. The construct was introduced into wild-type strain XS1150 by electroporation as described (Guerinot et al. 1990). Recombination was forced by selecting for the resistance to spectinomycin and for growth on 5% (w/v) sucrose. The mutant XS1150Δ*nopD* lacking the *nopD* gene was constructed in a similar procedure. The suicide vector contained the ΩSpe interposon with two flanking 1.3-kb sequences (DNA fragments upstream and downstream of the *nopD* coding sequence). Strain XS1150Δ*nopD*+*nopD* was constructed to rescue the XS1150Δ*nopD* mutant. The corresponding suicide vector contained a construct consisting of the *nopD* promoter region (1 kb), the coding region of *nopD* followed by a downstream sequence (1.4-kb) and a terminal Ωkm interposon (from the pET28b expression vector). Strain XS1150Δ*nopD*+*nopD*-C_{972}A was constructed using XS1150Δ*nopD* and a similar suicide vector construct that contained a mutation in *nopD* (encoding enzymatically inactive NopD-C_{972}A). Further details on plasmids and primers used for mutant construction are provided in Supplementary Tables S1 and S2.

Nodulation tests

Inoculation tests with *Bradyrhizobium* sp. XS1150 and peanut (*Arachis hypogaea*) plants indicated that this strain can induce nodules that promote plant growth. The legume *Tephrosia vogelii* was used for further nodulation tests with *Bradyrhizobium* sp. XS1150 and constructed mutant strains. Seeds were surface-sterilized and placed on 1% (w/v) water agar plates. After germination, seedlings were transferred to sterilized 300-ml plastic jar units (1 plant per jar). The units contained vermiculite and expanded clay granulates in the upper jar and nutrient solution containing 1 mM KNO_3 in the lower jar (Staehelin et al. 2006). Each plant was inoculated with a 2 ml-suspension (approximately 10^7 bacteria re-suspended in 10 mM $MgSO_4$). Plants were kept in an air-conditioned growth-room with a 16/8 h light/dark cycle at

24 ± 2 °C (ca. 2000 lux light intensity, Philips Lifemax TL-D 36W/54-765 and TL-D 36W/29-530 daylight fluorescent tubes at a ratio 3:1).

References

Bartsev, A. V., Deakin, W. J., Boukli, N. M., McAlvin, C. B., Stacey, G., Malnoë, P., et al. (2004). NopL, an effector protein of *Rhizobium* sp. NGR234, thwarts activation of plant defense reactions. Plant Physiol. 134, 871-879.

Beringer, J. E. (1974). R factor transfer in *Rhizobium leguminosarum*. J. Gen. Microbiol. 84, 188-198.

Broughton, W. J., Wong, C. H., Lewin, A., Samrey, U., Myint, H., Meyer, H. et al. (1986). Identification of *Rhizobium* plasmid sequences involved in recognition of *Psophocarpus*, *Vigna*, and other legumes. J. Cell Biol. 102, 1173-1182.

Guerinot, M. L., Morisseau, B. A., and Klapatch, T. (1990). Electroporation of *Bradyrhizobium japonicum*. Mol. Gen. Genet. 221, 287-290.

Hempel, J., Zehner, S., Göttfert, M., and Patschkowski, T. (2009). Analysis of the secretome of the soybean symbiont *Bradyrhizobium japonicum*. J. Biotechnol. 140, 51-58.

Marie, C., Deakin, W. J., Viprey, V., Kopciñska, J., Golinowski, W., Krishnan, H. B., et al. (2003). Characterization of Nops, nodulation outer proteins, secreted via the type III secretion system of NGR234. Mol. Plant-Microbe Interact. 16, 743-751.

Prentki, P., and Krisch, H. M. (1984). *In vitro* insertional mutagenesis with a selectable DNA fragment. Gene 29, 303-313.

Quandt, J., and Hynes, M. F. (1993). Versatile suicide vectors which allow direct selection for gene replacement in gram-negative bacteria. Gene 127, 15-21.

Sadowsky, M. J., Tully R. E., Cregan, P. B., Keyser, H. H. (1987). Genetic diversity in *Bradyrhizobium japonicum* serogroup 123 and its relation to genotype-specific nodulation of soybean. Appl. Environ. Microbiol. 53, 2624-2630.

Staehelin, C., Forsberg, L. S., D'Haeze, W., Gao, M. Y., Carlson, R. W., Xie, Z. P., et al. (2006). Exo-oligosaccharides of *Rhizobium* sp. strain NGR234 are required for symbiosis with various legumes. J. Bacteriol. 188, 6168-6178.

Vincent, J. M. (1970). A manual for the practical study of root-nodule bacteria. Blackwell, Oxford (UK).

Yoo, S. D., Cho, Y. H., and Sheen, J. (2007). Arabidopsis mesophyll protoplasts: a versatile cell system for transient gene expression analysis. Nat. Protoc 2, 1565-1572.

Zhang, S., and Liu, Y. (2001). Activation of salicylic acid-induced protein kinase, a mitogen-activated protein kinase, induces multiple defense responses in tobacco. Plant Cell 13, 1877-1889.

Zhang, L., Chen, X. J., Lu, H. B., Xie, Z. P., and Staehelin, C. (2011). Functional analysis of the type 3 effector nodulation outer protein L (NopL) from *Rhizobium* sp. NGR234: symbiotic effects, phosphorylation, and interference with mitogen-activated protein kinase signaling. J. Biol. Chem. 286, 32178-32187.
