# Supplementary material for: NopD of Bradyrhizobium sp. XS1150 Possesses SUMO Protease Activity
Source: Front Microbiol. 2020 Mar 20;11:386. doi: 10.3389/fmicb.2020.00386 (PMC7098955; doi:10.3389/fmicb.2020.00386)
Supplement: Supplementary file 2 [file Data_Sheet_2.PDF]

NopD of *Bradyrhizobium* sp. XS1150 possesses SUMO protease activity

Qi-Wang Xiang, Juan Bai, Jie Cai, Qin-Ying Huang, Yan Wang, Ying Liang, Zhi Zhong, Christian Wagner, Zhi-Ping Xie, and Christian Staehelin

Supplementary Table S1: Plasmids and strains used in this study

**Plasmids /Strains**	**Relevant Characteristics**	**Reference/Source**
pBluescript II SK (-)	High copy number ColE1-based phagemid, Amp^r	Stratagene (Agilent Technologies), Shanghai, China
Escherichia coli DH5α	*supE44* Δ*lac*U169 (*ϕ*80*lacZ*ΔM15) *hsdR17 recA1 endA1 gyrA96 thi-1 relA1*	GIBCO BRL, Bethesda, MD, USA
Escherichia coli BL21 (DE3)	F$^-$ *ompT hsdS$_B$* (r_B^- m_B^-) *gal dcm* (DE3)	Novagen (Merck Chemicals), Darmstadt, Germany
Bradyrhizobium sp. XS1150	A *Bradyrhizobium* sp. strain isolated from *Arachis hypogaea*; Cm^r	This study
pET28b	Expression vector for His-tag fusion proteins based on pBR322; Km^r	Novagen/Merck Chemicals (Darm-stadt, Germany)

pET28b-NopD	pET28b derivative carrying a *Nde*I-*Eco*RI fragment containing the coding region of *nopD* from *Bradyrhizobium* sp. XS1150 (accession number MF100854) fused to an N-terminal 6×His tag; PCR amplified from genomic DNA of XS1150 with primers 1 and 2; Km^r	This study
pET28b-NopD-C(640-1017)	pET28b derivative carrying a *Nde*I-*Eco*RI fragment encoding amino acid residues 640 to 1017 of NopD (accession number MF100854) fused to an N-terminal 6×His tag; PCR amplified from genomic DNA of XS1150 with primers 69 and 2; Km^r	This study
pET28b-NopD-C	pET28b derivative carrying a *Nde*I-*Eco*RI fragment encoding amino acid residues 721 to 1017 of NopD fused to an N-terminal 6×His tag, PCR amplified from pET28b-NopD with primers 2 and 6; Km^r	This study
pET28b-NopD-C_{972}A	pET28b derivative carrying a *Nde*I-*Eco*RI fragment encoding NopD of strain XS1150 with a C_{972}A substitution (cysteine residue 972 of NopD replaced by alanine) fused to an N-terminal 6×His tag, overlap extension PCR with primers 1, 2, 7 and 8; Km^r	This study
pET28b-XopD	pET28b derivative carrying a *Nde*I-*Bam*HI fragment containing the coding region of *xopD* of *Xanthomonas campestris* pv. *campestris* strain 8004 fused to an N-terminal 6×His tag, PCR amplified from genomic DNA with primers 9 and 10; Km^r	This study
pET28b-RanGAP-Myc	pET28b derivative carrying a *Bam*I-*Xho*I fragment containing the coding region of *RanGAP* of *Homo sapiens* (accession number NM_001317930.1) fused to an N-terminal 6×His tag and a C-terminal Myc tag; PCR amplified from cDNA of human liver (kindly provided by Qian Hu, Sun Yat-sen University) cDNA with primers 11 and 12; Km^r	This study

pET28b-AtSAE1	pET28b derivative carrying a *Sac*I-*Sal*I fragment containing the coding region of *AtSAE1* of *Arabidopsis thaliana* (accession number BT000094) fused to an N-terminal 6×His tag, PCR amplified from leaf cDNA of ecotype Columbia with primers 13 and 14; Km^r	This study
pET28b-AtUbc9	pET28b derivative carrying a *Sac*I-*Sal*I fragment containing the coding region of *AtUbc9* of *Arabidopsis thaliana* (accession NM_001202641) fused to an N-terminal 6×His tag, PCR amplified from leaf cDNA of ecotype Columbia with primers 15 and 16; Km^r	This study
pET28b-AtSAE2	pET28b derivative carrying a *Sac*I-*Sal*I fragment containing the coding region of *AtSAE2* of *Arabidopsis thaliana* (accession number BT003377) fused to an N-terminal 6×His tag; PCR amplified from leaf cDNA of ecotype Columbia with primers 17 and 18; Km^r	This study
pGEX-4T-1	Expression vector for GST-fusion proteins; Amp^r	Amersham Biosciences/GE Healthcare (Little Chalfont, UK)
pGEX-AtSUMO1-3HA	pGEX4T-1 derivative carrying a *Bam*HI-*Xho*I fragment containing the coding region of AtSUMO1-TGG of *Arabidopsis thaliana* (accession number AEE85259) fused to an N-terminal GST tag and a C-terminal 3HA tag; PCR amplified from leaf cDNA (ecotype Columbia) with primers 19, 20 and 21; Amp^r	This study
pGEX4T-AtSUMO-2-3HA	pGEX4T-1 derivative carrying an N-terminal GST tag fused to a *Bam*HI-*Xho*I fragment containing the coding region of AtSUMO2-TGG of *Arabidopsis thaliana* (accession	This study

	number NM_124898) with a C-terminal 3HA tag; PCR amplified from leaf cDNA (ecotype Columbia) with primers 21, 22 and 23; Amp^r	
pGEX4T-AtSUMO-3-3HA	pGEX4T-1 derivative carrying an N-terminal GST tag fused to a *Bam*HI-*Xho*I fragment containing the coding region of AtSUMO3-SGG of *Arabidopsis thaliana* (accession number NM_124899) with a C-terminal 3HA tag; PCR amplified from leaf cDNA (ecotype Columbia) with primers 21, 24 and 25; Amp^r	This study
pGEX4T-AtSUMO-5-3HA	pGEX4T-1 derivative carrying an N-terminal GST tag fused to a *Bam*HI-*Xho*I fragment containing the coding region of AtSUMO5-LGG of *Arabidopsis thaliana* (accession number NM_128836) with a C-terminal 3HA tag; PCR amplified from leaf cDNA (ecotype Columbia) with primers 21, 26 and 27; Amp^r	This study
pGEX4T-HuSUMO-1-3HA	pGEX4T-1 derivative carrying an N-terminal GST tag fused to a *Bam*HI-*Xho*I fragment containing the coding region of HuSUMO1-TGG of *Homo sapiens* (accession number AK311840) with a C-terminal 3HA tag; PCR amplified from cDNA of human liver (kindly provided by Qian Hu, Sun Yat-sen University) with primers 21, 28 and 29; Amp^r	This study
pGEX4T-HuSUMO-2-3HA	pGEX4T-1 derivative carrying an N-terminal GST tag fused to a *Bam*HI-*Xho*I fragment containing the coding region of HuSUMO2-TGG of *Homo sapiens* (accession number AK311837) with a C-terminal 3HA tag; PCR amplified from cDNA of human liver (kindly provided by Qian Hu, Sun Yat-sen University) with primers 21, 30 and 31; Amp^r	This study
pGEX4T-HuSUMO-4-3HA	pGEX4T-1 derivative carrying an N-terminal GST tag fused to a *Bam*HI-*Xho*I fragment	This study

	containing the coding region of HuSUMO4-TGG of *Homo sapiens* (accession number AB205057) with a C-terminal 3HA tag; PCR amplified from cDNA of human liver (kindly provided by Qian Hu, Sun Yat-sen University) with primers 21, 32 and 33; Amp^r	
pGEX4T-PvSUMO-3HA	pGEX4T-1 derivative carrying an N-terminal GST tag fused to a *Bam*HI-*Xho*I fragment containing the coding region of PvSUMO-TGG of *Phaseolus vulgaris* (accession number XM_007146455) with a C-terminal 3HA tag; PCR amplified from leaf cDNA of cv. Yudou No.1 with primers 21, 34 and 35; Amp^r	This study
pGEX4T-GmSUMO-3HA	pGEX4T-1 derivative carrying an N-terminal GST tag fused to a *Bam*HI-*Xho*I fragment containing the coding region of GmSUMO-TGG of *Glycine max* (accession number NM_001248279) with a C-terminal 3HA tag; PCR amplified from leaf cDNA of cv. Hefeng 45 with primers 21, 36 and 37; Amp^r	This study
pGEX4T-Smt3-3HA	pGEX4T-1 derivative carrying an N-terminal GST tag fused to a *BamH*I-*Xho*I fragment containing the coding region of SUMO-IGG of *Saccharomyces cerevisiae* (W303-1A) (accession number CP020194) with a C-terminal 3HA tag, PCR amplified from cDNA of *S. cerevisiae* with primers 21, 38 and 39; Amp^r	This study
pGEX-AtSUMO1(TGG)	pGEX4T-1 derivative carrying a *Bam*HI-*Eco*RI fragment containing the mature form of AtSUMO1 of *Arabidopsis thaliana* fused to an N-terminal GST tag; PCR amplified from pGEX4T-AtSUMO-1-3HA with primers 19 and 40; Amp^r	This study
pGEX-AtSUMO2(TGG)	pGEX4T-1 derivative carrying a *Bam*HI-*Eco*RI fragment containing the mature form of AtSUMO2 of *Arabidopsis thaliana* fused to an N-terminal GST tag; PCR amplified	This study

	from pGEX4T-AtSUMO-2-3HA with primers 22 and 41; Amp^r	
pGEX-AtSUMO3(SGG)	pGEX4T-1 derivative carrying a *Bam*HI-*Eco*RI fragment containing the mature form of AtSUMO3 of *Arabidopsis thaliana* fused to an N-terminal GST tag, PCR amplified from pGEX4T-AtSUMO-3-3HA with primers 24 and 42; Amp^r	This study
pGEX-AtSUMO5(LGG)	pGEX4T-1 derivative carrying a *Bam*HI-*Eco*RI fragment containing the mature form of AtSUMO5 of *Arabidopsis thaliana* fused to an N-terminal GST tag; PCR amplified from pGEX4T-AtSUMO-5-3HA with primers 26 and43; Amp^r	This study
pGEX-HuSUMO1(TGG)	pGEX4T-1 derivative carrying a *Bam*HI-*Eco*RI fragment containing the mature form of HuSUMO1 of *Homo sapiens* fused to an N-terminal GST tag; PCR amplified from pGEX4T-HuSUMO-1-3HA with primers 28 and 44; Amp^r	This study
pGEX-HuSUMO2(TGG)	pGEX4T-1 derivative carrying a *Bam*HI-*Eco*RI fragment containing the mature form of HuSUMO of *Homo sapiens* fused to an N-terminal GST tag; PCR amplified from pGEX4T-HuSUMO-2-3HA with primers 30 and 45; Amp^r	This study
pGEX-HuSUMO4(TGG)	pGEX4T-1 derivative carrying a *Bam*HI-*Eco*RI fragment containing the mature form of HuSUMO1 of *Homo sapiens* fused to an N-terminal GST tag; PCR amplified from pGEX4T-HuSUMO-4-3HA with primers 32 and 46; Amp^r	This study
pGEX-PvSUMO(TGG)	pGEX4T-1 derivative carrying a *Bam*HI-*Eco*RI fragment containing the mature form of PvSUMO of *Phaseolus vulgaris* cv. Yudou No.1 fused to an N-terminal GST tag; PCR amplified from pGEX4T-PvSUMO-3HA with primers 34and 47; Amp^r	This study

pGEX-GmSUMO(TGG)	pGEX4T-1 derivative carrying a *Bam*HI-*Eco*RI fragment containing the mature form of GmSUMO of *Glycine max cv.* Hefeng 45 fused to an N-terminal GST tag; PCR amplified from pGEX4T-GmSUMO-3HA with primers 36 and 48; Amp^r	This study
pGEX-Smt3(IGG)	pGEX4T-1 derivative carrying a *Bam*HI-*Eco*RI fragment containing the mature form of Smt3 of *Saccharomyces cerevisiae* (W303-1A) fused to an N-terminal GST tag; PCR amplified from pGEX4T-Smt3-3HA with primers 38 and 49; Amp^r	This study
pUC18-35S-MCS-polyA	pUC18 derivative carrying the CaMV 35S promoter, a multiple cloning site and a polyA fragment; Amp^r	Kindly provided by Jian Li, Laboratory of Prof. Nan Yao (Sun Yat-sen University, Guangzhou, China)
pA7-YFP	Transient expression vector suitable for particle bombardment; PUC18 derivative modified with a CaMV 35S promoter and an enhanced yellow fluorescent protein (eYFP); Amp^r	Kindly provided by Fang Wang, Laboratory of Prof. Da Luo (Sun Yat-sen University, Guangzhou, China)
pA7-NopD	pA7 derivative carrying an *Xho*I-*Spe*I fragment containing the coding region of *nopD* of *Bradyrhizobium* sp. XS1150 fused to a C-terminal YFP tag; PCR amplified from pET28b-NopD with primers 50 and 51; Amp^r	This study
pA7-NopD-C_{972}A	pA7 derivative carrying an *Xho*I-*Spe*I fragment encoding NopD of strain XS1150 with	This study

	a C972A substitution (cysteine 972 replaced by alanine) fused to a C-terminal YFP tag; PCR amplified from pET28b-NopD-C_{972}A with primers 50 and 51; Amp^r	
pA7-NopD-N	pA7 derivative carrying an *Xho*I-*Spe*I fragment encoding the N-terminal domain of NopD (amino acid residues 1 to 390) fused to a C-terminal YFP tag; PCR amplified from pET28b-NopD-N with primers 50 and 52; Amp^r	This study
pA7-NopD-NΔ2-53	pA7 derivative carrying an *Xho*I-*Spe*I fragment encoding methionine and amino acid residues 54 to 390 of NopD fused to a C-terminal YFP tag, PCR amplified from pET28b-NopD with primers 52 and 70; Amp^r	This study
pA7-NopD-NΔ2-60	pA7 derivative carrying an *Xho*I-*Spe*I fragment encoding methionine and amino acid residues 61 to 390 of NopD fused to a C-terminal YFP tag, PCR amplified from pET28b-NopD with primers 52 and 53; Amp^r	This study
pA7-NopD-C	pA7 derivative carrying an *Xho*I-*Spe*I fragment encoding amino acid residues 721 to 1017 of NopD fused to a C-terminal YFP tag; PCR amplified from pET28b-NopD with primers 51 and 54; Amp^r	This study
pA7-NopD-TR	pA7 derivative carrying an *Xho*I-*Spe*I fragment encoding the TR domain of NopD (amino acid residues 391 to 720) fused to a C-terminal YFP tag, PCR amplified from pET28b-NopD with primers 55 and 56; Amp^r	This study
pRT104	High copy vector carrying the CaMV 35S promoter, a polyadenylation signal and a modified polylinker of pUC18/19.	Töpfer et al. 1987

pRT104-NopD	pRT104 derivative carrying a *Nco*I-*Eco*RI fragment encoding NopD of *Bradyrhizobium* sp. XS1150 excised from pET28b-NopD; Amp^r	This study
pRT104-NopD-C_{972}A	pRT104 derivative carrying a *Nco*I-*Eco*RI fragment encoding NopD of XS1150 with a C972A substitution (cysteine 972 replaced by alanine) excised from pET28b-NopD-C_{972}A; Amp^r	This study
pCAMBIA1302	Binary vector with the CaMV 35S promoter and the coding sequence of *gfp*, Km^r	Cambia (Brisbane, Australia)
pCAMBIA1302-NopD	pCAMBIA1302 derivative carrying a *Hin*dIII fragment containing the 35S promoter and coding region of *nopD* excised from pRT104-NopD; Amp^r	This study
pCAMBIA1302-NopD-C_{972}A	pCAMBIA1302 derivative carrying a *Hin*dIII fragment containing the 35S promoter and DNA encoding NopD-C_{972}A excised from pRT104-NopD-C_{972}A; Amp^r	This study
Agrobacterium tumefaciens EH105	A hypervirulent *Agrobacterium tumefaciens* strain (Rif^r)	Gama et al. 1996
EH105 pCAMBIA1302	*A. tumefaciens* EH105 derivative carrying pCAMBIA1302, Rif^r, Amp^r	This study
EH105 pCAMBIA1302-NopT	*A. tumefaciens* EH105 derivative carrying pCAMBIA1302-NopT, Rif^r, Amp^r	Dai et al. 2008
EH105 pCAMBIA1302-NopD	*A. tumefaciens* EH105 derivative carrying pCAMBIA1302-NopD, Rif^r, Amp^r	This study
EH105 pCAMBIA1302-NopD-C_{972}A	*A. tumefaciens* EH105 derivative carrying pCAMBIA1302-NopD-C_{972}A, Rif^r, Amp^r	This study

pBS-*rhcST*	pBluescript II SK (-) derivative carrying a 1.5 kb *Sal*I-*Xba*I fragment containing the T3SS apparatus genes *rhcR, rhcS* and *rhcT* amplified from genomic DNA of *Bradyrhizobium* sp. strain XS1150 with primers 67 and 68; Amp^r	This study
pBS-2.6	pBluescript II SK (-) derivative with a 2.6-kb *Xba*I insert consisting of a 1.3-kb sequence upstream and a 1.3-kb sequence downstream of the coding sequence of *nopD*. The two sequences were PCR-amplified using genomic DNA of *Bradyrhizobium* sp. strain XS1150. An *Eco*RI restriction enzyme site was inserted in the middle of the fragment by overlap extension PCR with primers 57, 58, 59 and 60; Amp^r	This study
pBS-5.4	pBluescript II SK (-) derivative with a 5.4-kb *Xba*I fragment consisting of a 1.0-kb sequence upstream of *nopD*, the *nopD* coding sequence and a 1.4-kb sequence downstream of the coding sequence. The sequences were PCR amplified using genomic DNA of wild-type strain XS1150 as template. An *Eco*RI site was inserted at nucleotide position 400 downstream of the *nopD* coding sequence; construct made by overlap extension PCR with primers 61, 62, 63 and 64; Amp^r	This study
pBS-5.4-C_{972}A	pBluescript II SK (-)derived plasmid containing a 5.4-kb *Xba*I fragment consisting of a 1.0-kb sequence upstream of *nopD*, DNA encoding NopD-C_{972}-A (substitution of cysteine 972 to alanine) and a 1.4-kb sequence downstream of the coding sequence. The sequences were amplified from pBS-5.4. Cysteine residue 972 of NopD was replaced by alanine; construct made by overlap extension PCR with primers 7,8, 61 and 62; Amp^r	This study
pHP45Ω	Contains an ΩSpe interposon; Spe^r	Prentki and Krisch 1984

pBS-Ω*rhcST*	pBS-*rhcRST* derivative; a *Hin*dIII fragment containing the ΩSpe interposon from pHP45Ω was inserted into the *Hin*dIII site of pBS-*rhcRST*; Amp^r, Spe^r	This study
pBS-2.6-Ω	pBS-2.6 derivative; the Ωspe interposon from pHP45Ω was inserted into the created *Eco*RI site of pBS-2.6; Amp^r, Spe^r	This study
pBS-5.4-Ω	pBS-5.4 derivative; the 1.5-kb ΩKm interposon from pET28b was inserted into the created *Eco*RI site (see pBS-5.4); the interposon was PCR-amplified with primers 65 and 66; Amp^r, Km^r	This study
pBS-5.4-C_{972}A-Ω	pBS-5.4-C_{972}A derivative with the ΩKm interposon from pET28b inserted at the created *Eco*RI site (see pBS-5.4-C_{972}A); the interposon was PCR-amplified with primers 65and 66; Amp^r, Km^r	This study
pJQ200SK	A suicide vector containing a P15A origin of replication (*ori*), *lac*Zα system, a *sacB* gene for sucrose selection and a *mob* gene for mobilization, Gm^r	Quandt and Hynes 1993
pJQ-XS1150Ω*rhcST*	A derivative of pJQ200SK containing a 3.5 kb *Sal*I-*Xba*I fragment excised from pBS-Ω*rhcST*; Gm^r, Spe^r	This study
pJQ-XS1150Δ*nopD*	A derivative of pJQ200SK containing a 4.6-kb *Xba*I-*Xba*I fragment released from pBS-2.6-Ω with *Xba*I; Gm^r, Spe^r	This study
pJQ-XS1150*nopD*	A derivative of pJQ200SK containing a 6.9-kb *Xba*I-*Xba*I fragment released from pBS-5.4-Ω; Gm^r, Km^r	This study

pJQ-XS1150*nopD*-C_{972}A	A derivative of pJQ200SK containing a 6.9-kb *Xba*I-*Xba*I fragment released from pBS-5.4-C_{972}A-Ω; Gm^r, Km^r	This study
XS1150Ω*rhcST*	A T3SS-knockout mutant of *Bradyrhizobium* sp. strain XS1150 containing the Ωspe interposon inserted at the *Hin*dIII site of *rhcS*; constructed with pJQ-XS1150Ω*rhcST*; Spe^r	This study
XS1150Δ*nopD*	A *nopD* deletion mutant of *Bradyrhizobium* sp. strain XS1150; nucleotides 1-2974 of the *nopD* coding sequence were replaced by an Ωspe interposon; constructed with pJQ-XS1150Δ*nopD*; Cm^r, Spe^r	This study
XS1150Δ*nopD*+*nopD*	A derivative of XS1150Δ*nopD* with re-introduced *nopD*, the Ωspe interposon was replaced by the coding sequence of *nopD* and an Ωkm interposon was used as selection marker; constructed with pJQ-XS1150*nopD*; Cm^r, Km^r	This study
XS1150Δ*nopD*+*nopD-C_{972}A*	A point mutant of XS1150 producing NopD-C_{972}A; the Ωspe interposon of the XS1150Δ*nopD* mutant was replaced by DNA encoding NopD-C_{972}A and an Ωkm interposon was used as selection marker; constructed with pJQ-XS1150*nopD*-C972A; Cm^r, Km^r	This study
pBS-p*nopL*-N-E	pBluescript II SK (-) derivative containing the promoter and codon region of *nopL* from *Sinorhizobium* (*Ensifer*) sp. strain NGR234 containing *Nco*I and *Eco*RI restriction sites, PCR-amplified with primers 71-78: Amp^r	This study
pBS-NopD:NopL	A 150-bp *Nco*I-*Eco*RI fragment containing the 150-bp N-terminal secretion signal of NopD, PCR-amplified (primers 79 and 80) using genomic DNA of *Bradyrhizobium* sp.	This study

	XS1150 as template and cloned into pBS-pnopL-N-E digested with *Nco*I and *Eco*RI, Amp^r	
pFAJ1703	A stable RK2-derived cloning vector; Amp^r, Tc^r	Dombrecht et al. 2001
pFAJ-NopD:NopL	A 1.4-kb fragment containing the *nopL* promoter and the 150-bp secretion signal sequence of *nopD* fused to a *nopL* fragment encoding NopL lacking residues 2-50; excised from pBS-NopD:NopL with *Kpn*I and *Xba*I and cloned into pFAJ1703 digested with the same enzymes; Tc^r	This study
NGRΩ*nopL*	*Sinorhizobium* (*Ensifer*) sp. NGR234 derivative containing an Ω cassette inserted into the *Eco*RV site of the effector gene *nopL;* Rif^r; Km^r	Marie et al. 2003
NGRΩ*nopL* pFAJ-NopD:NopL	Mutant NGRΩ*nopL* carrying pFAJ-NopD:NopL; Rif^r, Km^r, Tc^r	This study
NGRΩ*rhcN*	*Sinorhizobium* (*Ensifer*) sp. NGR234 derivative containing an Ω cassette inserted into the *Eco*RI site of *rhcN* (no functional T3SS); Rif^r, Sp^r	Viprey et al. 1998
NGRΩ*rhcN* pFAJ-NopD:NopL	Mutant NGRΩ*rhcN* carrying pFAJ-NopD:NopL; Rif^r, Sp^r, Tc^r	This study

Abbreviations: Amp^r, Cm^r, Km^r, Rif^r, Spe^r, Tc^r, resistance to ampicillin, chloramphenicol, kanamycin, rifampin, spectinomycin and tetracycline, respectively.

References cited in Table S1

Dai, W. J., Zeng, Y., Xie, Z. P., and Staehelin, C. (2008). Symbiosis-promoting and deleterious effects of NopT, a novel type 3 effector of *Rhizobium* sp. strain NGR234. J. Bacteriol. 190, 5101-5110.

Dombrecht, B., Vanderleyden, J., and Michiels, J. (2001). Stable RK2-derived cloning vectors for the analysis of gene expression and gene function in gram-negative bacteria. Mol. Plant-Microbe Interact. 14, 426-30.

Gama, MICS., Leite, R. P., Cordeiro, A. R., and Cantliffe, D. J. (1996). Transgenic sweet potato plants obtained by *Agrobacterium tumefaciens*-mediated transformation. Plant Cell, Tissue and Organ Culture 46, 237-244.

Marie, C., Deakin, W. J., Viprey, V., Kopciñska, J., Golinowski, W., Krishnan, H. B., Perret, X., and Broughton, W. J. (2003). Characterization of Nops, nodulation outer proteins, secreted via the type III secretion system of NGR234. Mol. Plant-Microbe Interact. 16, 743-751.

Prentki, P., and Krisch, H. M. (1984). *In vitro* insertional mutagenesis with a selectable DNA fragment. Gene 29, 293-303.

Quandt, J., and Hynes, M. F. (1993). Versatile suicide vectors which allow direct selection for gene replacement in Gram-negative bacteria. Gene 127, 15-21.

Töpfer, R., Matzeit, V., Gronenborn, B., Schell, J., and Steinbiss, H. H. (1987). A set of plant expression vectors for transcriptional and translational fusions. Nucleic Acids Res. 15, 5890.

Viprey, V., Greco, A. D., Golinowski, W., Broughton, W. J., and Perret, X. (1998). Symbiotic implications of type III protein secretion machinery in *Rhizobium*. Mol. Microbiol. 28, 1381-1389.
