# Supplementary material for: NopD of Bradyrhizobium sp. XS1150 Possesses SUMO Protease Activity
Source: Front Microbiol. 2020 Mar 20;11:386. doi: 10.3389/fmicb.2020.00386 (PMC7098955; doi:10.3389/fmicb.2020.00386)
Supplement: Supplementary file 3 [file Data_Sheet_3.PDF]

NopD of *Bradyrhizobium* sp. XS1150 possesses SUMO protease activity

Qi-Wang Xiang, Juan Bai, Jie Cai, Qin-Ying Huang, Yan Wang, Ying Liang, Zhi Zhong, Christian Wagner, Zhi-Ping Xie, and Christian Staehelin

Supplementary Table S2. Primers used for plasmid construction.

No	Sequence (5' to 3')	Restriction site	Description
1	GGAATTCCATATGGTGGACCCGTACAAT	*Nde*I	For construction of pET28b-NopD
2	ATAGAATCCTCAGTTGAAGCCGAC	*Eco*RI	
6	GGAATTCCATATGGCCGGTAAACCCGC	*Nde*I	For construction of pET28b-NopD-C
7	ACAGTTATGACGCCGGCGTCTTTGTC		For construction of pET28b-NopD-C972A
8	GACAAAGACGCCGGCGTCATAACTGT		
9	GCGGCAGCCATATGGTGGAATCCCAAGAC	*Nde*I	For construction of pET28b-XopD
10	CGCGGATCCCTAGAACTTTTTCCAC	*Bam*HI	
11	GCCGGATCCGATGGCCTCGGAAGAC	*Bam*HI	For construction of pET28b-RanGAP-Myc
12	CCGCTCGAGTTAGAGGTCTTCTTCGGAAATCAACTTCTGTTC GACCTTGTACAGCGT	*Xho*I	

13	ATTGAGCTCCATGGACGGAGAAGAG	*Sac*I	For construction of pET28b-AtSAE1
14	ATTGTCGACTTAAGAGGTAAAAGAG	*Sal*I	
15	ATTGAGCTCCATGGCTACGCAACAAC	*Sac*I	For construction of pET28b-AtUbc9
16	ATTGTCGACCTATTCAACTCTTAT	*Sal*I	
17	ATTGAGCTCCATGGCTAGTGGAATC	*Sac*I	For construction of pET28b-AtSAE2
18	ATTGTCGACTTAGGCATAATCCGCGACGTCATACGGGTAGAC<br>AAGAGCAGGAT	*Sal*I	
19	GCCGGATCCATGTCTGCAAACCAG	*Bam*HI	For construction of pGEX-AtSUMO1-3HA
20	TACGTCGTATGGGTAGCCACCAGTCTGATG		
21	CCGCTCGAGTTAGGCATAATCCGGGACGTCATACGGGTATG<br>CATAGTCAGGAACATCATAAGGATAAGCGTAATCTGGTACG<br>TCGTATGGGTA	*Xho*I	
22	GCCGGATCCATGTCTGCTACTCC	*Bam*HI	For construction of pGEX-AtSUMO2-3HA
23	TACGTCGTATGGGT ACCACCAGTCTGAT		
24	GCCGGATCCATGTCTAACCCTCAAG	*Bam*HI	For construction of pGEX-AtSUMO3-3HA
25	TACGTCGTATGGGT ACCACCACTCATCG		
26	GCCGGATCC ATGGTGAGTTCCACAG	*Bam*HI	For construction of pGEX-AtSUMO5-3HA
27	TACGTCGTATGGGT ACCACCAAGTTCCAT		
28	GCCGGATCC ATGTCTGACCAGGAG	*Bam*HI	For construction of pGEX-HuSUMO1-3HA

29	TACGTCGTATGGGT ACCCCCCGTTTGTT		
30	GCCGGATCC ATGGCCGACGAAAAG	*Bam*HI	For construction of pGEX-HuSUMO2-3HA
31	TACGTCGTATGGGT ACCTCCCGTCTGCTG		
32	GCCGGATCC ATGGCCAACGAAAAG	*Bam*HI	For construction of pGEX-HuSUMO4-3HA
33	TACGTCGTATGGGT ACCTCCCGTAGGCTGT		
34	GCCGGATCCATGTCGGGCGTTACT	*Bam*HI	For construction of pGEX4T-PvSUMO-3HA
35	TACGTCGTATGGGTACCACCAGTCTGATG		
36	GCCGGATCC ATGTCGGGCGTTACC	*Bam*HI	For construction of pGEX4T-GmSUMO-3HA
37	TACGTCGTATGGGT ACCTCCTGTCTGGTG		
38	GCCGGATCC ATGTCGGACTCAGAAG	*Bam*HI	For construction of pGEX4T-Smt3-3HA
39	TACGTCGTATGGGTACCACCAATCTGTTCT		
40	CCGCTCGAGTCAGCCACCAGTCTGATGG	*Xho*I	For construction of pGEX-AtSUMO1(TGG)
41	CCGCTCGAGTCAACCACCAGTCTGAT	*Xho*I	For construction of pGEX-AtSUMO2(TGG)
42	CCGCTCGAGTCAACCACCACTCATCG	*Xho*I	For construction of pGEX-AtSUMO3(SGG)
43	CCGCTCGAGTCAACCACCAAGTTCCAT	*Xho*I	For construction of pGEX-AtSUMO5(LGG)
44	CCGCTCGAGTCAACCCCCCGTTTGTT	*Xho*I	For construction of pGEX-HuSUMO1(TGG)

45	CCGCTCGAGTCAACCTCCCGTCTGCTG	*Xho*I	For construction of pGEX-HuSUMO2(TGG)
46	CCGCTCGAGTCAACCTCCCGTAGGCTGT	*Xho*I	For construction of pGEX-HuSUMO4(TGG)
47	CCGCTCGAGTCAACCACCAGTCTGATG	*Xho*I	For construction of pGEX-PvSUMO(TGG)
48	CCGCTCGAGTCAACCTCCTGTCTGGTG	*Xho*I	For construction of pGEX-GmSUMO(TGG)
49	CCGCTCGAGTCAACCACCAATCTGTTCT	*Xho*I	For construction of pGEX-Smt3(IGG)
50	CCGCTCGAGATGGACCCGTACAATTTC	*Xho*I	For construction of pA7-NopD and pA7-NopD-C_{972}A
51	CGGACTAGTGTTGAAGCCGACAC	*Spe*I	
52	CGGACTAGTCACCGCTTCCGGCGT	*Spe*I	For construction of pA7-NopD-N
53	CCGCTCGAGATGCTCTCTGCAGAACAT	*Xho*I	For construction of pA7-NopD-NΔ2-60
54	CCGCTCGAGATGGCCGGTAAACCCGCG	*Xho*I	For construction of pA7-NopD-C
55	CCGCTCGAGATGCCAGATGGGTCGTCG	*Xho*I	For construction of pA7-NopD-TR
56	CGGACTAGTAGGGCTGAGCACCGG	*Spe*I	
57	TGCTCTAGATTCATGATCTCGCGT	*Xba*I	For construction of pBS-2.6
58	TGCTCTAGAGAACCACAGCAGTCAG	*Xba*I	
59	GGACGTGAGGCTATCGAATTCGGACCTGAACCTCAAC	*Eco*RI	

60	GTTGAGGTTCAGGTCCGAATTCGATAGCCTCACGTCC	*Eco*RI	
61	TGCTCTAGAACACGAAATCGCTGT	*Xba*I	For construction of pBS-5.4 and pBS-5.4-C_{972}A
62	TGCTCTAGAACCCAATCGTTGAC	*Xba*I	
63	GCGCCTTCCCAAGACGAATTCCTGTCAATGCGCGCT	*Eco*RI	
64	AGCGCGCATTGACAGGAATTCGTCTTGGGAAGGCGC	*Eco*RI	
65	CCGGAATTCAGCGTCAATTTGTTT	*EcoR*I	For construction of pBS-5.4 and pBS-5.4-C_{972}A-Ω
66	CCGGAATTCTATGTAGGCGGTGCT	*EcoR*I	
67	GTTGTTCTNTTCCTCGTSCGC*		For construction of pBS-Ω*rhcST*
68	ATTCTTGATCRBCGGRGASAG*		
69	GGAATTCCATATGCACATCTACCGCGAT	*Nde*I	For construction of pET28b-NopD-C(640-1017)
70	CCGCTCGAGATGGGCCGCAACTACCAT	*Xho*I	For construction of pA7-NopD-NΔ2-53
71	GTGGTACCAGAAGAAATATCGTGTTG	*Kpn*I	For construction of pBS-p*nopL*-N-E
72	TCGGATCCTCAAATGTCAAAATCC	*Bam*HI	
73	TCAAAGGAGAAACACCATGGATATCAATTC	*Nco*I	
74	AGTTTCCTCTTTGTGGTACCTATAGTTAAG		
75	CCGCAGGTTGAAGCCATGAATTCCCCATATTT	*Eco*RI	
76	GGCGTCCAACTTCGGTACTTAAGGGGTATAAA		
77	CACGTTGCCGAATTTCGAGGCGAGGGAGT		
78	GTGCAACGGCTTAAAGCTCCGCTCCCTCA		

79	ATTCCATGGACCCGTACAATTTCGAT	*Nco*I	For construction of pBS-NopD:NopL
80	GGGGAATTCATGACAGGACCCGGATCGG	*Eco*RI	

*Abbreviations: N = A/G/C/T; B = G/C/T; R = A/G; S = G/C; W = A/T.
