# Supplementary material for: NopD of Bradyrhizobium sp. XS1150 Possesses SUMO Protease Activity
Source: Front Microbiol. 2020 Mar 20;11:386. doi: 10.3389/fmicb.2020.00386 (PMC7098955; doi:10.3389/fmicb.2020.00386)
Supplement: Supplementary file 4 [file Data_Sheet_4.PDF]

NopD of *Bradyrhizobium* sp. XS1150 possesses SUMO protease activity

Qi-Wang Xiang, Juan Bai, Jie Cai, Qin-Ying Huang,Yan Wang, Ying Liang, Zhi Zhong,
Christian Wagner, Zhi-Ping Xie, and Christian Staehelin

Supplementary Figures

This file contains:

Supplementary Fig. S1. Schematic representation of constructed mutants in the *nopD* gene of *Bradyrhizobium* sp. XS1150.
Supplementary Fig. S2. *Bradyrhizobium* sp. XS1150 induces nodules on *T. vogelii.*
Supplementary Fig. S3. The sequenced T3SS gene cluster of *Bradyrhizobium* sp. XS1150.
Supplementary Fig. S4. Alignment of a conserved C-terminal region of NopD with related proteins.
Supplementary Fig. S5. Expression of a C-terminal NopD fragment in *E. coli* and analysis of the prepared anti-NopD antibody.
Supplementary Fig. S6. Preparation of various SUMO-RanGAP conjugates *in vitro*.
Supplementary Fig. S7. Subcellular localization of additional NopD variants in plant cells.
Supplementary Fig. S8. Alignment of SUMO protein sequences used in this study.

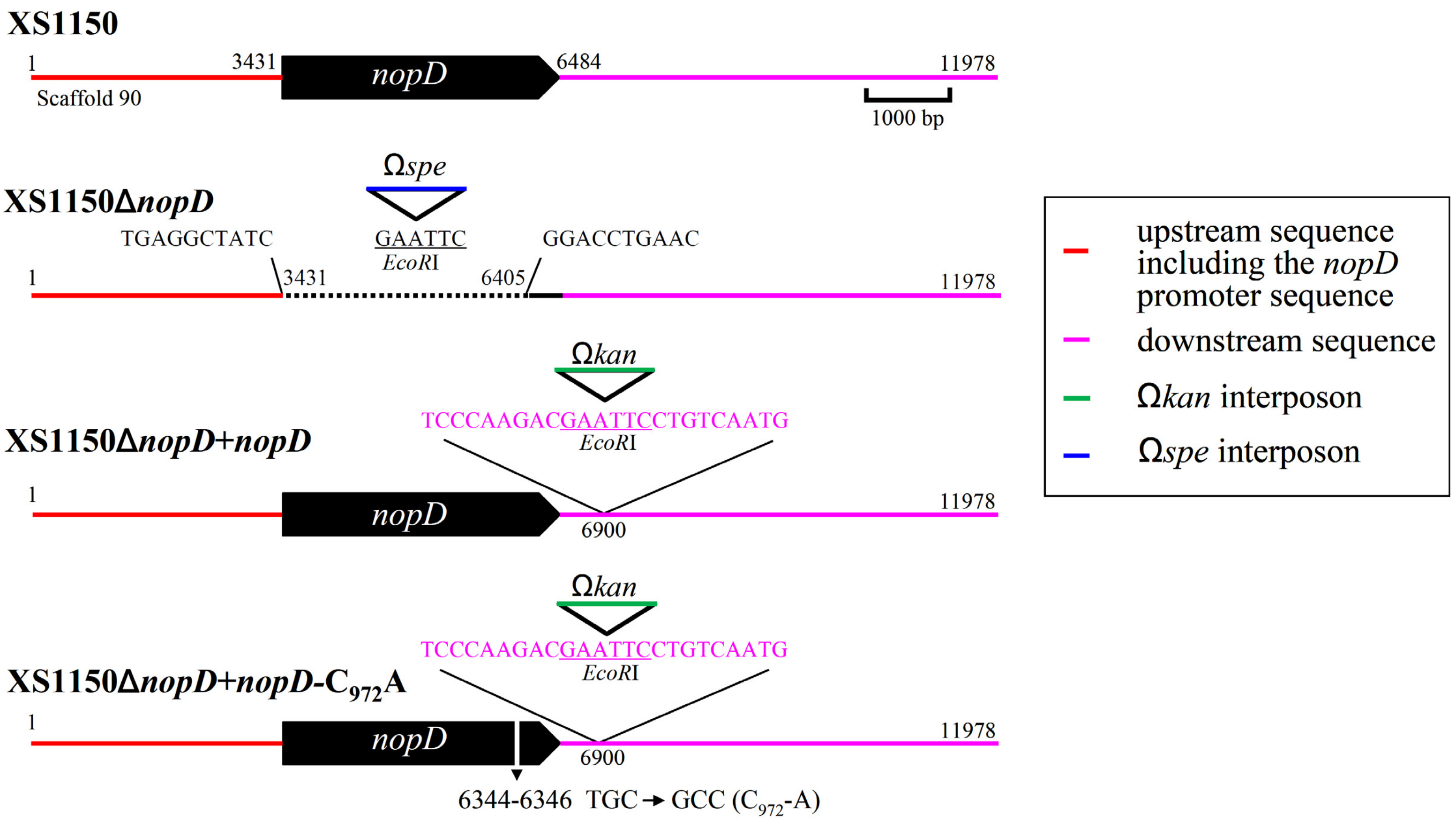

Supplementary Fig. S1. Schematic representation of constructed mutants in the *nopD* gene of *Bradyrhizobium* sp. XS1150. Details for mutant construction are mentioned in Supplementary Text 1.

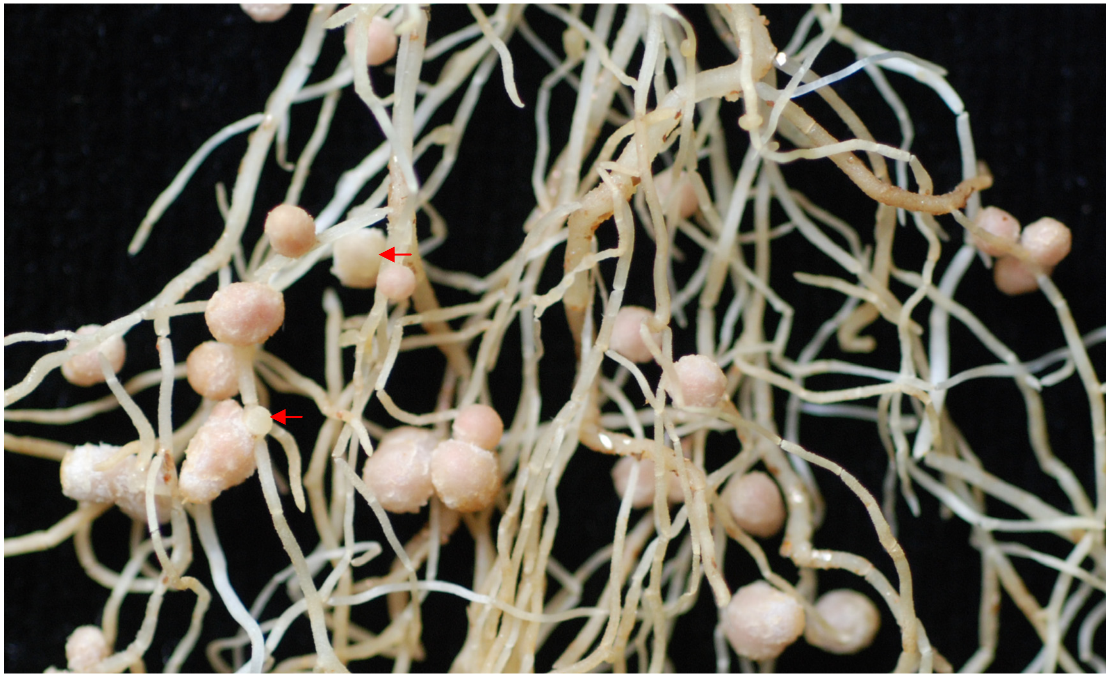

Supplementary Fig. S2. *Bradyrhizobium* sp. XS1150 induces nodules on *T. vogelii*. Plants mock-inoculated with 10 mM $MgSO_4$ did not form any nodules. The picture shows an example for a nodulated root system (plants were harvested 36 days post inoculation). Most nodules were pink (expression of leghemoglobin). White nodules (no or low leghemoglobin expression) were also observed (arrows).

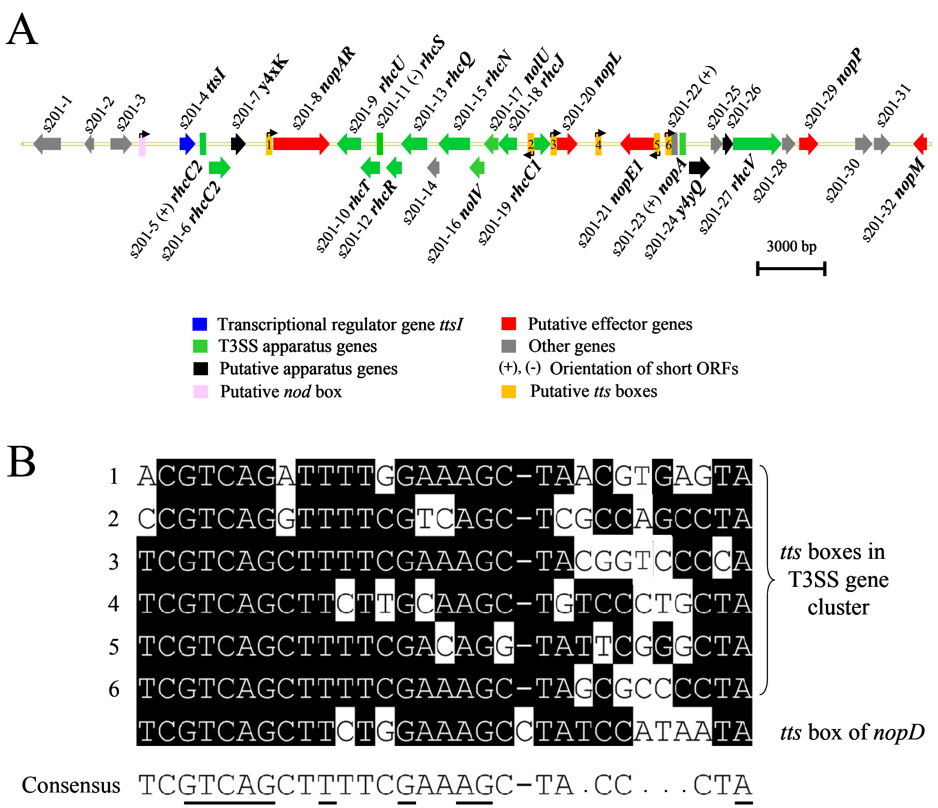

Supplementary Fig. S3. The sequenced T3SS gene cluster of *Bradyrhizobium* sp. XS1150 **(A)** A complete T3SS gene cluster was identified on scaffold 201 of the draft genome of strain XS1150 (accession number NFUH01000201.1). The cluster includes T3SS apparatus genes, the transcriptional regulator gene *ttsI* and putative effector genes (*nopAR*, *nopL, nopE1*, *nopP* and a fragment of *nopM*). Gene prediction was performed with the prodigal.v2_60 software. **(B)** Alignment of the putative *tts* boxes in the T3SS gene cluster of scaffold 201 (*tts* box 1, nucleotides 10871-10900; *tts* box 2, 22226 to 22197; *tts* box 3, 23079 to 23108; *tts* box 4, 25115 to 25144; *tts* box 5, 27756 to 27727; and *tts* box 6, 28330 to 28359). A putative *tts* box was also found in the promoter region of *nopD* in scaffold 90 (accession number NFUH01000090.1; nucleotides 3363 to 3393). Underlined nucleotide sequences in the consensus sequence indicate conserved nucleotides as identified by alignment of *tts* boxes from various rhizobial strains (Krause et al. 2002, Mol. Plant-Microbe Interact. 15, 1228-1235).

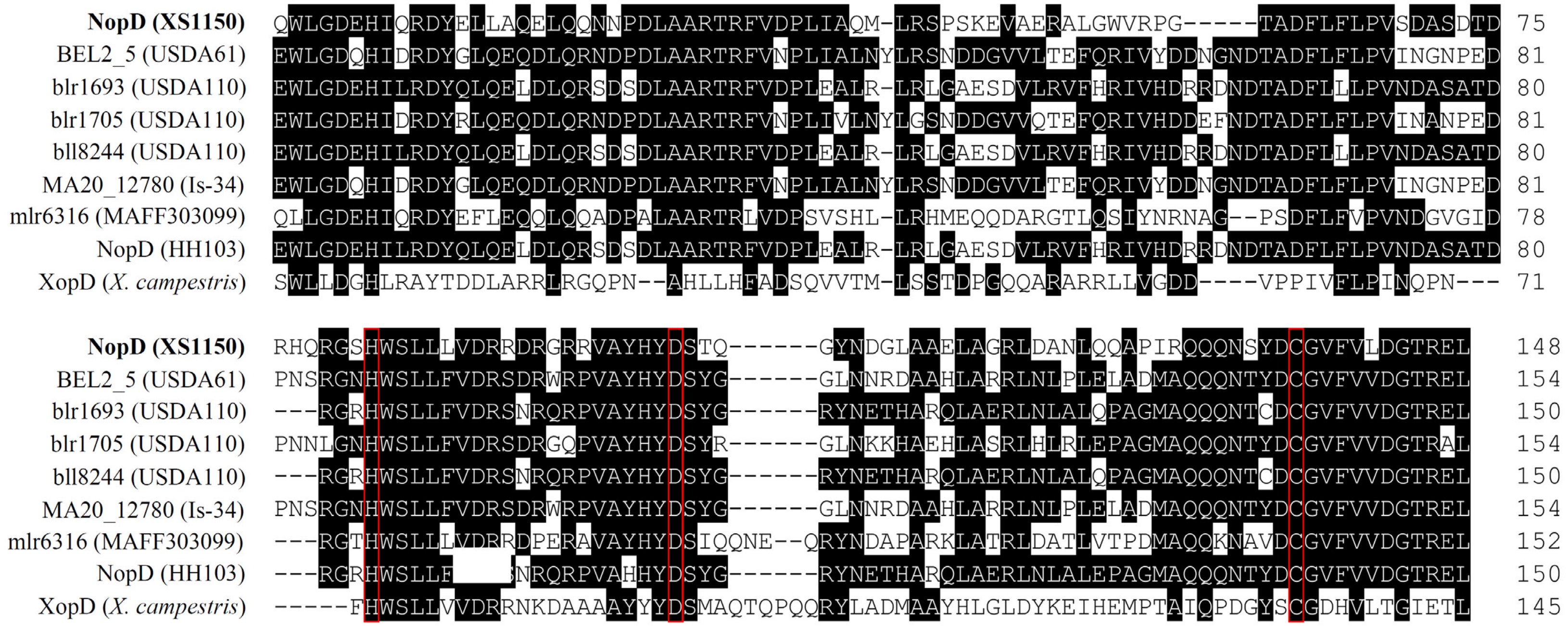

Supplementary Fig. S4. Alignment of a conserved C-terminal region of NopD with related proteins. The C-terminal part of NopD (shown are the most conserved residues 835-982) possesses sequence similarities with various proteins of the genera *Bradyrhizobium*, *Mesorhizobium* and *Sinorhizobium*. The sequence of the SUMO protease XopD (*Xanthomonas* T3 effector) was included into the alignment. Residues of the conserved catalytic triad of SUMO proteases (C48 cysteine peptidases) are framed in red. Sequence accession numbers: MF100854 (NopD of *Bradyrhizobium* sp. XS1150, residues 835-982); AKS25901 (BEL2_5 of *B. elkanii* USDA61, residues 1144-1297), KGT79298 (MA20_12780 of *B. japonicum* Is-34, residues 1180-1333), BAC53509 (bll8244 of *B. japonicum* USDA110, residues 1261-1410), BAC46970 (blr1705 of *B. japonicum* USDA110, residues 1259-1412), BAC46970 (blr1693 of *B. japonicum* USDA110, residues 1536-1685), BAB52630 (mlr6316 of *Mesorhizobium loti* MAFF303099, residues 1430-1581), CCE98838 (NopD of *Sinorhizobium fredii* HH103, residues 1138-1287), and WP_011038021 (XopD of *Xanthomonas campestris* pv. campestris 8004, residues 222-366).

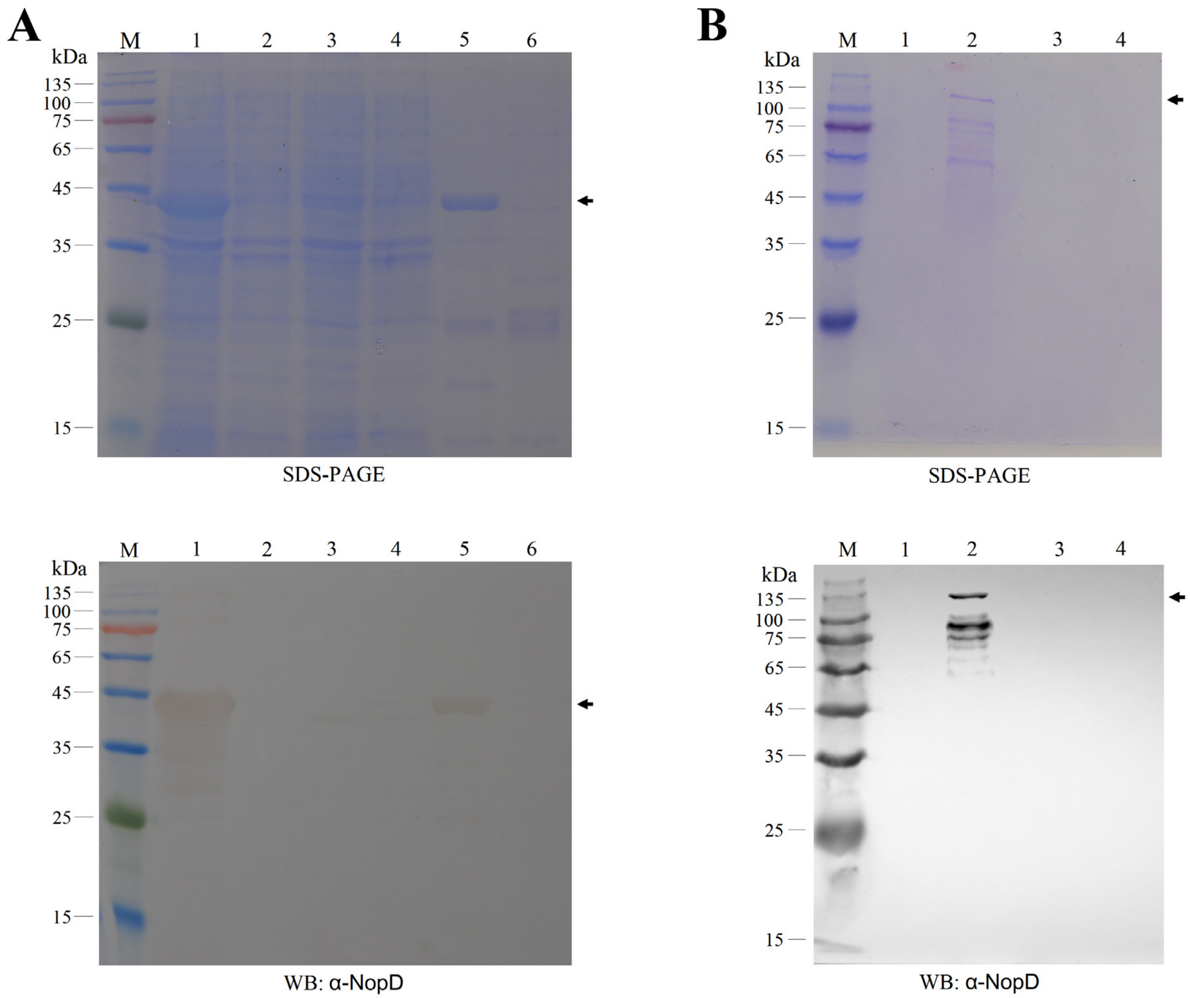

Supplementary Fig. S5. Expression of NopD in *E. coli*. Proteins were subjected to SDS-PAGE (top) and Western blot analysis (bottom). Protein expression in *E. coli* BL21 (DE3) was induced by 0.5 mM isopropyl-β-D-thiogalactopyranosid (IPTG) and cultures were kept at 18°C for 20 hours. **(A)** Analysis of the 6×His-tagged NopD fragment (residues 640-1017) used for

production of an anti-NopD antibody. Western blot analysis was performed with a 1:5000 dilution of the antibody and 3,3'-diaminobenzidine. Lane M, molecular weight marker. Lane 1, proteins from cells expressing the NopD fragment after IPTG induction. Lane 2, proteins from IPTG-treated cells carrying the empty vector pET28. Lane 3, proteins of the strain expressing the NopD fragment grown in the absence of IPTG. Lane 4, proteins from cells carrying the empty vector pET28 without IPTG treatment. Lane 5, the Ni-NTA purified NopD fragment (arrow) from IPTG-treated cells. Lane 6, corresponding control proteins from IPTG-treated cells carrying the empty vector. **(B)** Analysis of 6×His-tagged full-length NopD. Proteins were analyzed after Ni-NTA purification. Western blot analysis was performed with the anti-NopD antibody (1:3000 dilution) and electrochemiluminescence detection reagents. Lane M, molecular weight marker. Lane 1, proteins from cells carrying the pET28b-NopD plasmid grown in the absence of IPTG. Lane 2, purified NopD (arrow) from IPTG-induced cells. The lower bands likely represent degraded NopD forms. Lane 3, control proteins from cells carrying the empty vector grown in the absence of IPTG. Lane 4, control proteins from IPTG-treated cells carrying the empty vector.

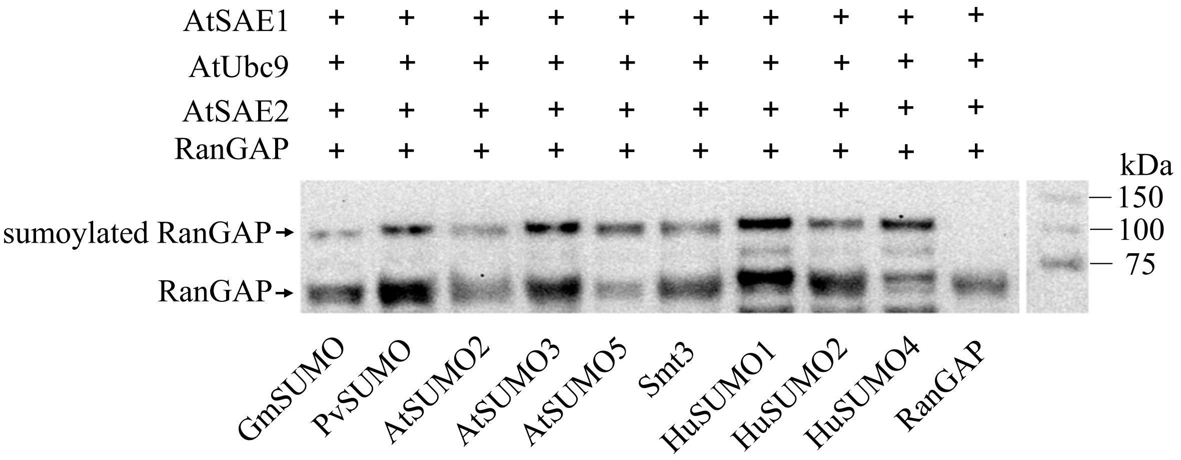

Supplementary Fig. S6. Preparation of various SUMO-RanGAP conjugates *in vitro*. The sumoylation systems contained indicated 6×His-tagged *Arabidopsis* proteins, human RanGAP (with 6×His and Myc tags) and GST-tagged SUMO proteins from soybean (GmSUMO), *P. vulgaris* (PvSUMO), *Arabidopsis* (AtSUMO3, AtSUMO2, and AtSUMO5), yeast (Smt3) or human (HuSUMO1, HuSUMO2 and HuSUMO4). Detection of the formed SUMO-RanGAP conjugates and RanGAP on the Western blot was performed with an anti-Myc antibody.

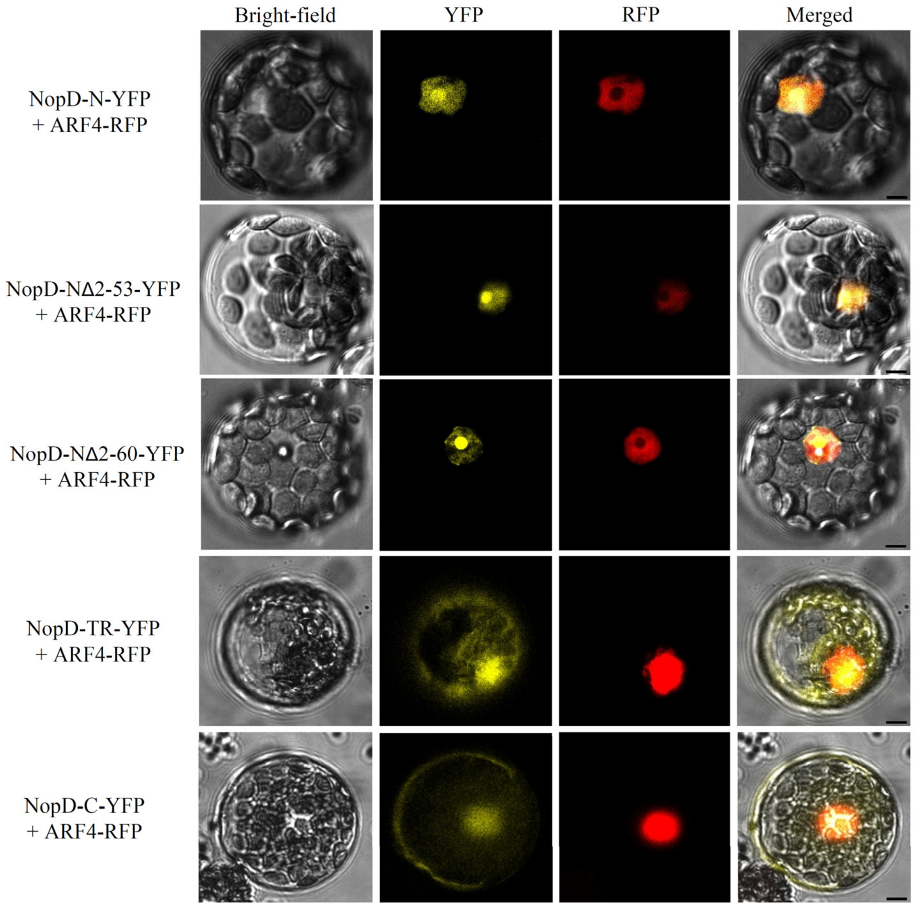

Supplementary Fig. S7. Subcellular localization of additional NopD variants in plant cells. Indicated NopD variants fused to YFP were expressed in *Arabidopsis* protoplasts. The nuclear marker ARF4 (auxin response factor 4 of *A. thaliana*) fused to RFP was co-expressed. Cells were analyzed with a confocal microscope for yellow fluorescence (YFP) emission, red fluorescence (RFP) emission, and under bright-field illumination (18 h after transformation). Bars: 5 µm.

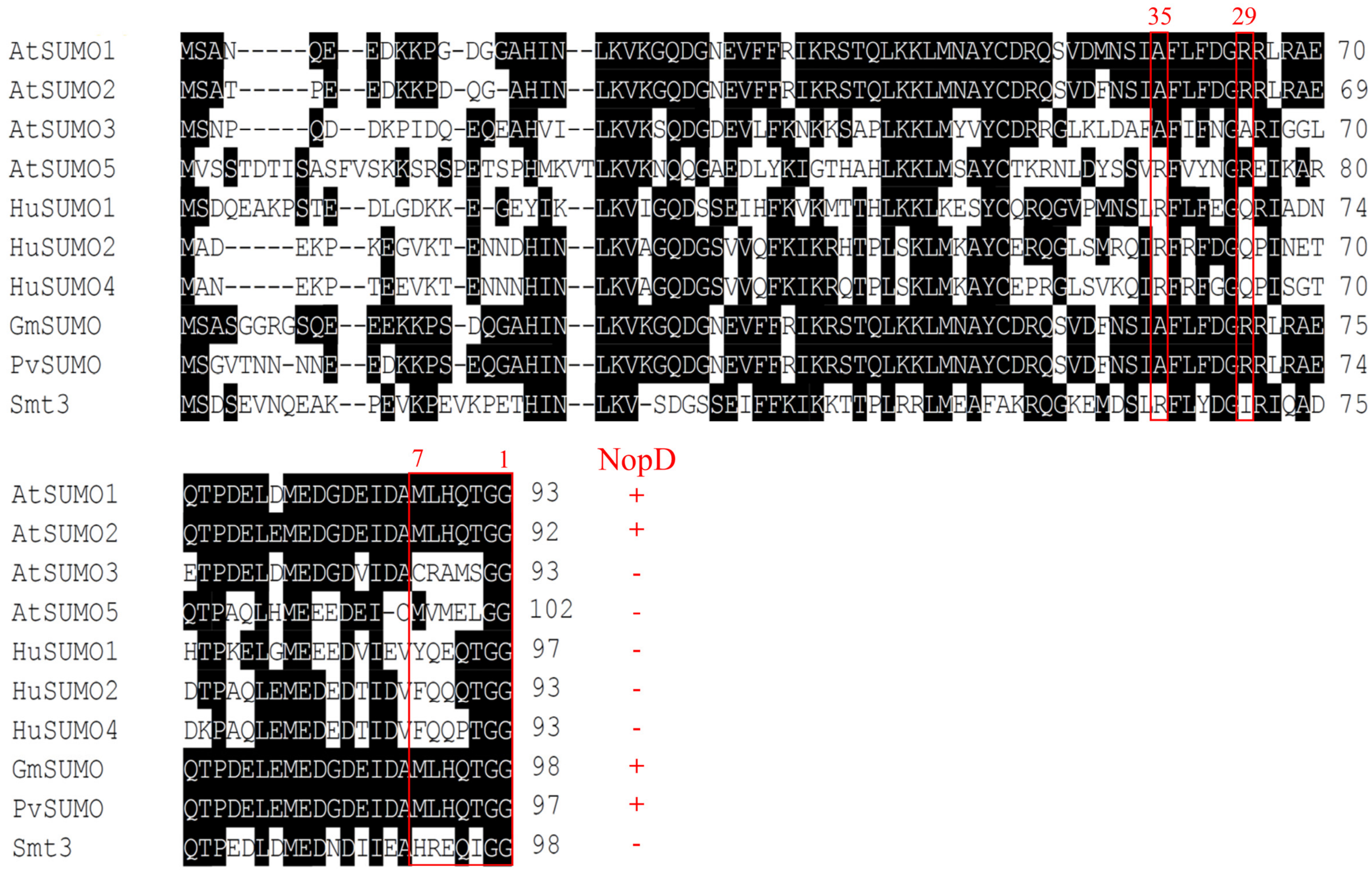

Supplementary Fig. S8. Alignment of SUMO protein sequences used in this study. Only the processed forms with the C-terminal di-glycine motif are shown. NopD of *Bradyrhizobium* sp. XS1150 could cleave AtSUMO1, AtSUMO2, GmSUMO and PvSUMO. These SUMO proteins possess the conserved recognition motif A_{35}-R_{29}-$M_7L_6H_5Q_4T_3G_2G_1$ (positions of amino acid residues are framed).
