# Supplementary material for: NopD of Bradyrhizobium sp. XS1150 Possesses SUMO Protease Activity
Source: Front Microbiol. 2020 Mar 20;11:386. doi: 10.3389/fmicb.2020.00386 (PMC7098955; doi:10.3389/fmicb.2020.00386)
Supplement: Supplementary file 5 [file Data_Sheet_5.PDF]

NopD of *Bradyrhizobium* sp. XS1150 possesses SUMO protease activity

Qi-Wang Xiang, Juan Bai, Jie Cai, Qin-Ying Huang, Yan Wang, Ying Liang, Zhi Zhong,
Christian Wagner, Zhi-Ping Xie, and Christian Staehelin

Non-cropped images of Figures 1-3 and S6

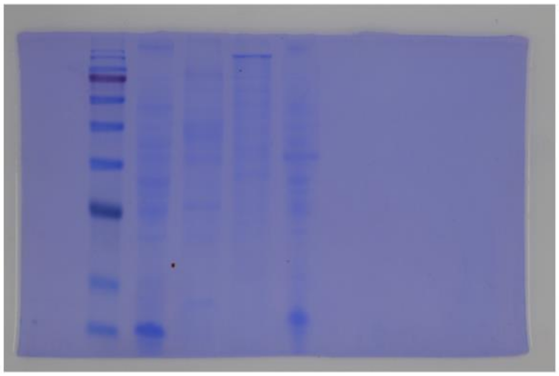

SDS-PAGE

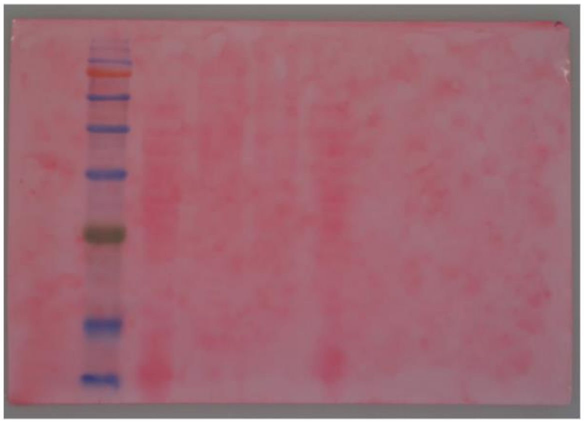

Ponceau staining

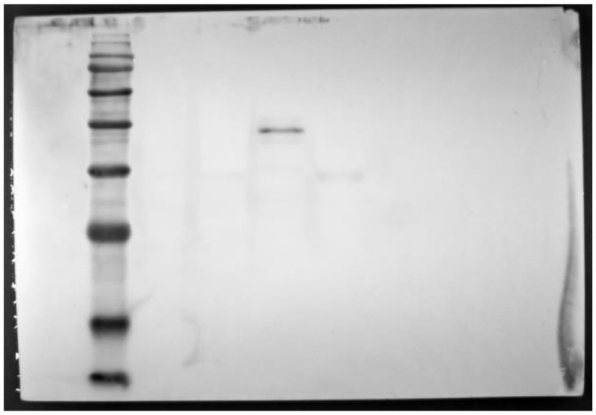

Western blot

Non-cropped images of Fig. 1c

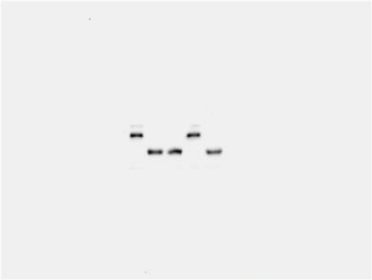

GST-AtSUMO1

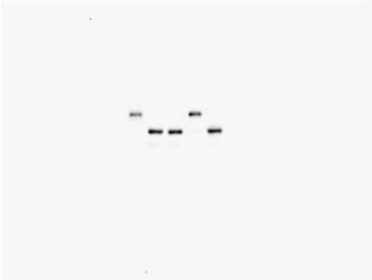

GST-AtSUMO2

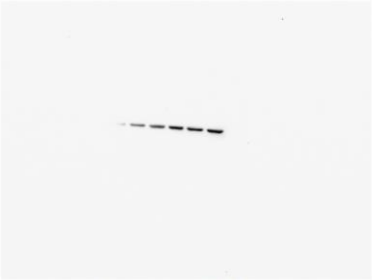

GST-AtSUMO3

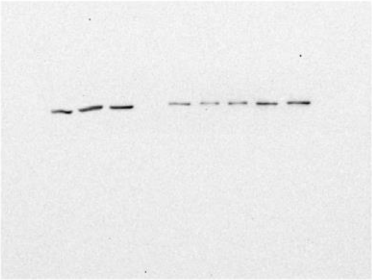

GST-AtSUMO5

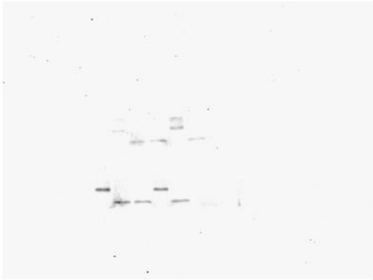

GST-GmSUMO

GST-HuSUMO1

GST-HuSUMO2

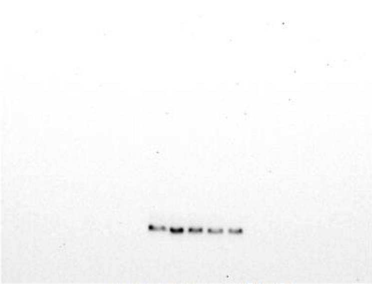

GST-HuSUMO4

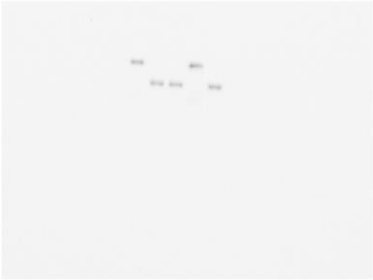

GST-PvSUMO

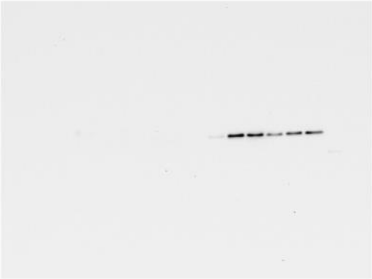

GST-Smt3

Non-cropped images of Fig. 2b and 2c

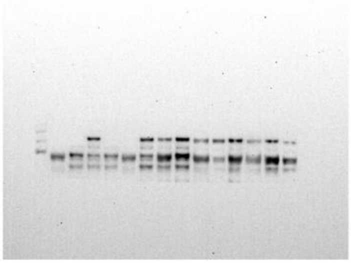

Fig. 3A

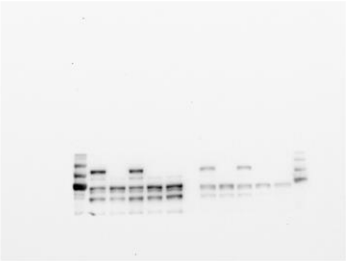

Fig. 3B GmSUMO and AtSUMO1

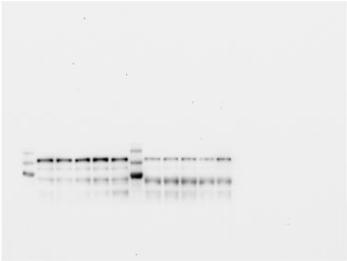

Fig. 3C HuSUMO1 and AtSUMO5

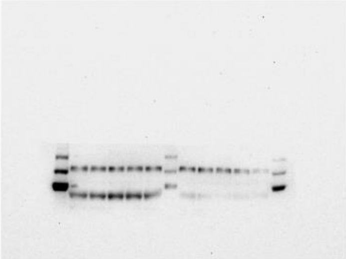

Fig. 3C HuSUMO2 and AtSUMO3

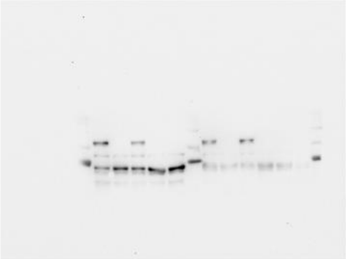

Fig. 3B PvSUMO and AtSUMO2

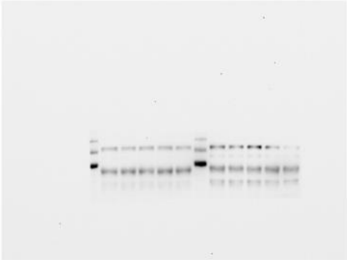

Fig. 3C HuSUMO4 and Smt3

Non-cropped images of Fig. 3

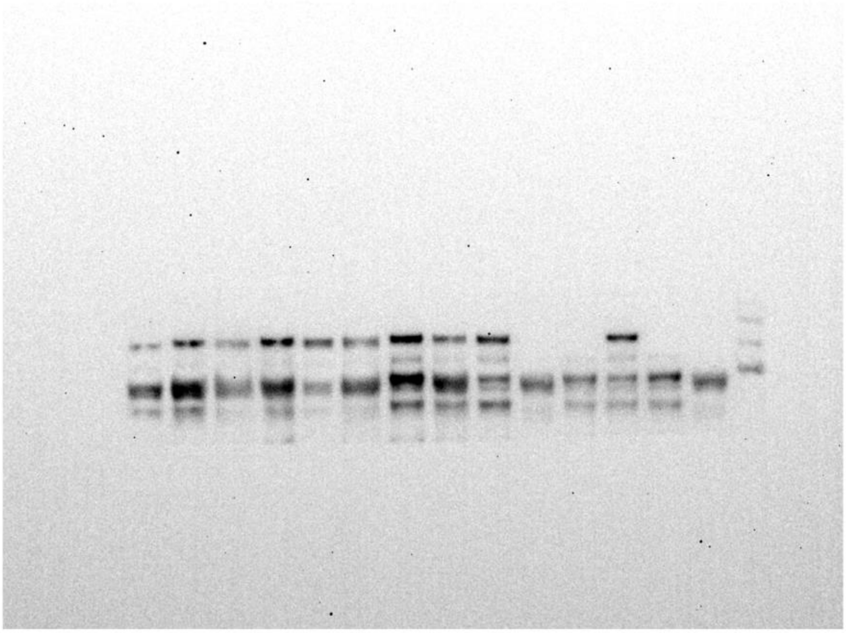

Non-cropped image of Fig. S6
